# Burden of sickle cell anemia in Africa: A systematic review and meta-analysis

**DOI:** 10.1371/journal.pone.0337090

**Published:** 2025-11-25

**Authors:** Bwambale Jonani, Emmanuel Charles Kasule, Bwire Roman Herman, Joel Fredrick Arturo, Mwesigwa Calvin Mugambwa, Ssebulime Stephen, John Bosco Mundaka, Richard Kwizera, Gerald Mboowa, Felix Bongomin

**Affiliations:** 1 Department of Clinical Laboratories, Sebbi Hospital, Wakiso, Uganda; 2 Department of Immunology and Molecular Biology, School of Biomedical Sciences, College of Health Sciences, Makerere University, Kampala, Uganda; 3 Outpatient and Inpatient Departments, Sebbi Hospital, Wakiso, Uganda; 4 Department of Obstetrics & Gynecology, Sebbi Hospital, Wakiso, Uganda; 5 Department of Research, Infectious Diseases Institute, College of Health Sciences, Makerere University, Kampala, Uganda; 6 The African Centre of Excellence in Bioinformatics and Data-Intensive Sciences, Infectious Diseases Institute, Makerere University, Kampala, Uganda; 7 Department of Medical Microbiology and Immunology, Faculty of Medicine, Gulu University, Gulu, Uganda; University of North Carolina at Chapel Hill, UNITED STATES OF AMERICA

## Abstract

**Introduction:**

Sickle Cell Anemia (SCA) is a significant genetic disorder in Africa; however, comprehensive data on its prevalence and geographic distribution remain limited. We aimed to estimate the pooled prevalence of SCA (HbSS) in African populations and examine regional, demographic, and temporal variations from 1994–2024.

**Methods:**

We systematically searched PubMed, Scopus, Google Scholar, and BASE databases for studies reporting SCA prevalence in African populations. Screening and quality assessments were performed using JBI tools. A random-effects meta-analysis with logit transformation was performed, with subgroup analyses by region, age, sex, and study design. Meta-regression explored heterogeneity sources, including geographic region, age category, diagnostic method, study design, and publication year.

**Results:**

From 115 studies with 1,203,839 participants and 17,458 confirmed HbSS cases, the pooled prevalence was 1.43% (95% CI: 1.08%–1.88%), with substantial heterogeneity (I^2^ = 99.1%) and a prediction interval of 0.21%–8.91%. Central Africa showed the highest prevalence (1.99%), and Southern Africa showed the lowest (0.59%). Children exhibited a higher prevalence (1.65%) than adults (0.45%), while sex differences were non-significant (males 2.71%, females 1.74%; p = 0.694). The prevalence has remained stable over three decades despite a six-fold increase in research output, although wide prediction intervals indicated substantial between-study variability. Electrophoretic techniques predominated (86.4% of cases). Diagnostic method (χ² = 16.73, p = 0.033) and age category (χ² = 33.66, p < 0.0001) significantly moderated the prevalence. The multivariable meta-regression was marginally significant (χ² = 29.01, p = 0.066), but substantial residual heterogeneity persisted (I^2^ = 98.6%). Leave-one-out sensitivity analysis showed that no single study significantly impacted the pooled estimates.

**Conclusion:**

SCA represents a substantial and geographically variable public health challenge across Africa. These findings highlight the need for region-specific interventions, expanded newborn screening programs, improved diagnostic accessibility with quality assurance for point-of-care technologies, and continued surveillance to address geographic gaps.

## Introduction

Sickle cell anemia (SCA) is an autosomal recessive genetic disorder characterized by abnormal hemoglobin S in red blood cells, causing them to sickle when deoxygenated [1–3]. Severe forms include hemoglobin SS (sickle-cell anemia) from homozygous HbS inheritance and HbS/β^o^ thalassemia from co-inheritance of HbS with a β^o^ thalassemia mutation, where individuals produce no normal beta-globin [1,4,5]. Other variations involve co-inheritance of HbS with different β-globin gene mutations, such as hemoglobin C, hemoglobin D-Los Angeles/Punjab, or β+ thalassemia, which results in reduced beta-globin production [4,5].

Between 2000 and 2021, worldwide SCA diagnoses increased by 382 per 100,000 live births, rising from 453,000–515,000 [6]. The genotype distribution was as follows: 76.5% SS and Sβ° births, 19.6% SC births, and 3.9% Sβ + . Globally, SCA mortality has increased, with sub-Saharan Africa experiencing the highest rates [7]. In 2021, 29,400 individuals died from SCA in this region, a 30.1% increase from 2000, representing 2.2% of child mortality and the 11^th^ leading cause of all-cause mortality [5,6].

Over the last three decades, research on SCA in Africa has advanced significantly. Newborn screening programs have expanded, enabling early diagnosis and interventions [8,9]. Point-of-care test kits have enhanced early detection [10–12], and treatments such as hydroxyurea have improved the quality of life of patients with SCA [13,14]. Genetic research has revealed the critical role of fetal hemoglobin (HbF) in reducing disease severity [15–17]. Public health initiatives have recognized SCA as a major concern leading to the establishement of research and healthcare networks to improve patient outcomes.

Despite these advancements, challenges remain. Many African countries have not consistently implemented neonatal screening or preventive measures for sickle cell anemia (SCA), which hinders early diagnosis and timely intervention. This is due to a lack of reliable data on the prevalence of SCA and the distribution of hemoglobin types across regions, making it difficult to accurately estimate the disease burden in the country. Child mortality rates in Africa remain among the highest globally, with approximately 50–90% of children dying before the age of five [18,19]. This is partly due to inadequate healthcare infrastructure, delayed diagnoses, cultural stigma, and limited awareness of SCA.

Although the burden of SCA in Africa is widely acknowledged [6,20], previous epidemiological estimates are fragmented or limited to specific regions, populations, and age groups [4,18]. Several country-level studies and regional summaries exist [9,21,22], but no recent systematic review has comprehensively synthesized prevalence data across all African regions, accounting for diagnostic methods, demographic subgroups and temporal trends. This lack of robust aggregated data impedes the development of evidence-based screening programs, resource allocation, and policy formulation. In light of the increased diagnostic capacity and research output in Africa since the 1990s, an updated systematic review and meta-analysis was needed to consolidate the burden of SCA on a continental scale and identify persistent gaps in surveillance and care delivery.

This review aimed to estimate the pooled prevalence of genetically confirmed sickle cell anemia (HbSS) in the African population between 1994 and 2024. We further sought to assess how prevalence varies by region, age group, sex, diagnostic method, and study design, and to evaluate temporal trends and methodological heterogeneity among studies.

## Materials and methods

### Study design

This quantitative systematic review was performed and reported according to the Preferred Reporting Items for Systematic Reviews and Meta-Analyses (PRISMA) guidelines (S1 Table). The protocol for this meta-analysis was registered with the Open Science Framework (OSF) on January 8, 2025 (https://doi.org/10.17605/OSF.IO/M8JXV) and published in PLOS ONE [23]. No amendments were made to the protocol after its registration.

### Eligibility criteria

We included observational studies (cross-sectional and cohort) that reported the prevalence of sickle cell anemia (SCA) in African populations, specifically defined by the presence of the homozygous hemoglobin SS (HbSS) genotype. Studies were eligible if they were published between January 1, 1994, and December 31, 2024. Only studies using validated diagnostic methods, including hemoglobin electrophoresis, high-performance liquid chromatography (HPLC), isoelectric focusing, solubility tests, point-of-care tests (e.g., HemotypeSC™ or SickleScan), or DNA-based methods, were considered. We excluded case reports, reviews, editorials, conference abstracts lacking primary data, and interventional studies, such as randomized controlled trials, that did not report SCA prevalence. Studies that lacked confirmed diagnostic criteria or reported only sickle cell traits (HbAS) without distinction from SCA were also excluded. For subgroup analyses, studies were grouped by geographic region (North, West, Central, East, and Southern Africa), age category (newborns, children, adults, mixed), sex, study design, and diagnostic method.

### Information sources

We searched four major databases: PubMed, Scopus, Google Scholar, and BASE (Bielefeld Academic Search Engine). We also manually screened the reference lists of all the included articles for additional studies. Grey literature (e.g., theses) was considered when full texts were accessible. No language restrictions were imposed. The last database search was conducted on January 15, 2025. These databases were selected for their broad and complementary coverage of biomedical and global academic literature, particularly in public health, hematology, and African studies fields.

### Search strategy

A comprehensive search strategy was developed using Medical Subject Headings (MeSH) and free-text terms. Boolean operators (AND, OR) were used to combine terms related to sickle cell anemia, prevalence, and African countries. No date or language filters were applied to the search. The full search string used in PubMed was: “Sickle Cell Disease”[All Fields] OR “Sickle Cell Anaemia”[All Fields] OR “Sickle Cell”[All Fields] AND (“prevalence”[All Fields] OR “epidemiology”[All Fields] OR “burden”[All Fields]) AND (“Africa”[All Fields] OR “Uganda”[All Fields] OR “Nigeria”[All Fields] OR “Kenya”[All Fields] OR “Ghana”[All Fields] OR “South Africa”[All Fields] OR “Tanzania”[All Fields] OR “Ethiopia”[All Fields] OR “Democratic Republic of Congo”[All Fields] OR “Cameroon”[All Fields] OR “Ivory Coast”[All Fields] OR “Senegal”[All Fields] OR “Sudan”[All Fields] OR “Zambia”[All Fields] OR “Zimbabwe”[All Fields] OR “Malawi”[All Fields] OR “Mozambique”[All Fields] OR “Burkina Faso”[All Fields] OR “Mali”[All Fields] OR “Sierra Leone”[All Fields] OR “Benin”[All Fields] OR “Botswana”[All Fields] OR “Burundi”[All Fields] OR “Cape Verde”[All Fields] OR “Central African Republic”[All Fields] OR “Chad”[All Fields] OR “Comoros”[All Fields] OR “Djibouti”[All Fields] OR “Equatorial Guinea”[All Fields] OR “Eritrea”[All Fields] OR “Eswatini”[All Fields] OR “Gabon”[All Fields] OR “Gambia”[All Fields] OR “Guinea”[All Fields] OR “Guinea-Bissau”[All Fields] OR “Lesotho”[All Fields] OR “Liberia”[All Fields] OR “Madagascar”[All Fields] OR “Mauritania”[All Fields] OR “Mauritius”[All Fields] OR “Namibia”[All Fields] OR “Niger”[All Fields] OR “Rwanda”[All Fields] OR “Sao Tome and Principe”[All Fields] OR “Seychelles”[All Fields] OR “Somalia”[All Fields] OR “South Sudan”[All Fields] OR “Togo”[All Fields] OR “Western Sahara”[All Fields] OR “Algeria”[All Fields] OR “Angola”[All Fields] OR “Egypt”[All Fields] OR “Libya”[All Fields] OR “Morocco”[All Fields] OR “Tunisia”[All Fields]).

The search strategies for Scopus, Google Scholar, and BASE followed the same logic and used equivalent terms and Boolean combinations (S2 Table).

### Selection process

Three reviewers (BJ, ECK, and HRB) independently screened all titles and abstracts for eligibility based on the inclusion and exclusion criteria. The full texts of potentially relevant studies were retrieved and assessed independently. Discrepancies were resolved through discussion or, if necessary, arbitration by a fourth reviewer (JBM and SS). Screening was performed manually, without the use of automation tools.

### Data collection process

Data were extracted independently by two reviewers (JB and ECK) using a standardized extraction form that was piloted before use. The collected data included the author, publication year, country, study design, population characteristics (sample size and age category), diagnostic method, and number of confirmed HbSS cases. The study authors were not contacted for missing or unclear data due to feasibility constraints. No automation tools were employed.

### Data items

#### Outcomes.

The primary outcome was the **prevalence of sickle cell anemia (HbSS)**, defined as the proportion of individuals with confirmed HbSS in the study population. Where multiple measures were reported (e.g., by sex and age), all relevant results were extracted. If subgroup data were reported without an overall prevalence, we calculated an aggregate if the raw data were available.

#### Other variables.

Other extracted variables included the study setting (hospital vs. community-based), diagnostic method, age category (newborn, child, adult, mixed), region (North, East, West, Central, and Southern Africa), year of data collection, and study design. When classification was ambiguous (e.g., age categories spanning child and adult), categorization was based on the median age or dominant age group.

### Risk of bias assessment

The quality of each included study was evaluated using the Joanna Briggs Institute (JBI) Critical Appraisal Checklist for Prevalence Studies. This tool evaluated nine domains related to methodological rigor and potential sources of bias. These included the appropriateness of the sample frame, suitability of the sampling method, adequacy of the sample size, clarity in describing the study population and setting, and coverage of a representative portion of the sample in the data analysis. The tool also assessed the use of valid and reliable diagnostic methods, consistency in measuring the condition across all participants, appropriate statistical analyses, and adequate management of response rates if they were low. Additionally, we explicitly evaluated whether the methods used to ascertain outcomes, specifically the diagnostic approaches for confirming SCA, were described clearly and consistently applied across participants. To assess potential reporting bias, we examined whether the studies presented multiple measurements or analyses of the same outcome, as this could indicate selective reporting of only favorable results. A study was considered to have a low risk of bias if it met at least eight of the nine JBI criteria and demonstrated valid diagnostic methods, consistent outcome measurements, and proper handling of response rates. A moderate-to-low risk of bias was assigned to studies that met at least six criteria and fulfilled key methodological expectations. Studies that failed to meet at least six criteria or had major flaws in outcome ascertainment, measurement consistency, or response rate handling were considered to have a high risk of bias. Only studies with a low or moderate risk of bias were included in the final meta-analysis to ensure the validity and reliability of findings.

### Effect measures

The effect measure used was the pooled prevalence proportion, with corresponding 95% confidence intervals (CIs). Raw prevalence data were transformed using logit transformation to stabilize the variance before the meta-analysis. For studies reporting zero events (0% prevalence), we applied a continuity correction of 0.5 to both the numerator and denominator to enable logit transformation.

### Synthesis methods

#### Study grouping.

The studies were grouped by age category, sex, region, diagnostic method, and study design to allow for subgroup and sensitivity analyses. The age categories included newborns (typically 0–28 days), children (generally encompassing individuals from 1 month to 17 years), and adults (≥ 18 years). When age distributions spanned more than one category or were reported as broad population groups, classification was based on the predominant age range or the median age of participants.

#### Data preparation.

All prevalence data were standardized to proportions. Subgroup data were aggregated where necessary. Geographic regions were standardized to the WHO African regions (North, West, Central, East, or Southern Africa). For the temporal analysis, a yearly dataset was generated by identifying all the unique years represented in the data. For each year, pooled prevalence estimates and corresponding confidence intervals were calculated. For studies reporting data collection across multiple years, we assigned a midpoint year. For retrospective studies spanning extended periods, we used the final year of the data collection. For prospective cohorts, we used the enrollment year. This approach balanced temporal precision with practical constraints, as most studies did not report exact monthly data collection periods. The results were merged with a complete set of years covering the study period to ensure continuity in the timeline, even when no data were available for specific years. Diagnostic methods for confirming Sickle Cell Anemia were grouped into five broader methodological categories to enhance consistency across studies: Rapid Sickling Tests (point-of-care or biochemical tests such as solubility or sickling assays), Chromatographic Methods (including High-Performance Liquid Chromatography), Electrophoretic Techniques (such as hemoglobin electrophoresis and isoelectric focusing), Molecular Methods (including DNA-based techniques),

#### Data presentation.

Summary characteristics were tabulated, and the results were displayed using forest plots, funnel plots, and PRISMA flow diagrams.

#### Meta-analysis and models.

We used the metaprop() function from the R Meta package to perform a random-effects meta-analysis. Statistical heterogeneity was assessed using Cochran’s Q and I^2^ statistics. Analyses were conducted in R version 4.5.1.

#### Exploration of heterogeneity.

To identify the sources of heterogeneity, we conducted subgroup analyses and meta-regressions using moderators, including region, diagnostic method, age category, and study design.

#### Sensitivity analyses.

We performed a leave-one-out sensitivity analysis to assess the impact of each individual study on the overall pooled prevalence by sequentially removing each study, recalculating the pooled estimate, and measuring the absolute difference and percentage change from the overall pooled effect. Studies were flagged as influential if their absolute differences exceeded a threshold of 0.002. The 75th percentile of the percentage change was calculated to identify studies with a larger impact on the meta-analysis results. A revised meta-analysis was subsequently conducted without the influential studies to compare the new pooled estimate with the original. The overlap of confidence intervals (CIs) from both analyses was examined, with substantial shifts indicated by non-overlapping CIs, suggesting a significant influence. A table was generated to visually represent the pooled prevalence of sickle cell anemia, showing the impact of each study’s removal.

#### Reporting bias assessment.

We assessed publication bias using Egger’s regression test and visual inspection of funnel plots. No significant asymmetry was observed.

#### Certainty of evidence.

We assessed the overall quality of the cumulative evidence using AMSTAR (A Measurement Tool to Assess systematic Reviews) tool.

## Results

### Search results

**Fig 1:**
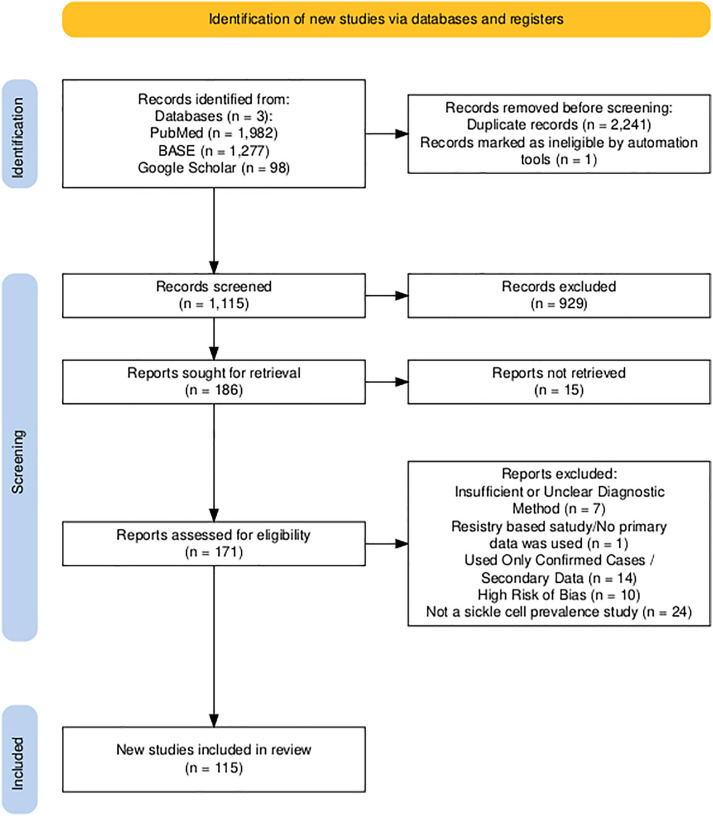
PRISMA flow diagram demonstrating the study selection process.

The search yielded 3,614 records screened. After removing duplicates, 1,115 records remained for screening purposes. Independent review of the titles and abstracts of these records by four reviewers resulted in Fleiss’ Kappa value of 0.546 (z = 44.3, p < .0001), prompting a joint screening of the records, which resulted in 186 studies being selected as eligible. The full texts of 15 studies were not available after multiple search efforts and attempts to request them from the authors. Following a detailed full-text review and quality appraisal, 56 studies were excluded based on one or more of the following criteria: insufficient or unclear diagnostic method, registry-based/no primary data, high risk of bias, and not being a sickle cell prevalence study (S3 Table). A total of 115 studies were included in the final analysis (Fig 1).

### Synthesis overview

We included 115 studies with 1,203,839 participants and 17,458 confirmed HbSS cases. Of the 115 included studies, most were cross-sectional (n = 72), retrospective (n = 22), or prospective (n = 16), with a minority comprising quasi-experimental designs (n = 1), case-control (n = 2), controlled trial (n = 1), or mixed study design (n = 1) (Table 1). All studies used laboratory-confirmed diagnostic methods. The majority (n = 105, 91.3%) were rated as having a low risk of bias (S4 Table). These studies spanned 28 countries across Africa.

**Table 1:** Characteristics of included studies.

Author, reference	Year	Country	Study Design	Sample Size	Age Category	HBSS Cases	Diagnostic Method
Abdala et al [24]	2024	Republic of Congo	Cross sectional Study	448	Children	57	HemotypeSC™
Abdulhamid et al [25]	2024	Nigeria	Cross sectional Study	2478	Adults	2	PCR
Adegoke [26]	2024	Gambia	Cross sectional Study	1168	New born	15	HemotypeSC™
Asare et al [27]	2024	Ghana	Cross sectional Study	504	Mixed Age groups	1	HB Electrophoresis
Brahim et al [28]	2024	Mauritania	Cross sectional Study	565	Adults	50	HB Electrophoresis
Gomez et al [29]	2024	Benin	Cross sectional Study	2910	Children	91	HPLC
Kiyaga Charles [30]	2024	Uganda	Retrospective Study	12009	Children	63	IsoElectric Focussing
Mano et al [31]	2024	Namibia	Cross sectional Study	202	New born	0	HemotypeSC™
Namukasa et al [32]	2024	Uganda	Cross sectional Study	399	Adults	3	HB Electrophoresis
Olaniyan et al [33]	2023	Angola	Retrospective Study	2000	New born	34	IsoElectric Focussing
Mumbere et al [34]	2023	Democratic Republic of Congo	Cross sectional Study	1195	New born	2	HemotypeSC™
Nyangasa et al [35]	2023	Tanzania	Prospective Study	152	Mixed Age groups	9	HPLC
Olewe et al [36]	2023	Kenya	Cross sectional Study	79	Children	0	PCR
Tchum et al [37]	2023	Ghana	Randomised Control Trial	860	Children	4	PCR
Tutuba et al [38]	2023	Tanzania	Quasi Experimental design	247	Mixed Age groups	4	Sickle scan (POCT)
Chao et al [39]	2022	Nigeria	Retrospective Study	11142	Children	97	Sickle scan
Christopher et al [40]	2022	Tanzania	Cross sectional Study	676	New born	3	IsoElectric Focussing
Dokekias et al [41]	2022	Republic of Congo	Cross sectional Study	782	Adults	14	Emmel test
Kambale Kombi et al [42]	2022	Democratic Republic of Congo	Cross sectional Study	221	Adults	0	Mass spectrormoetry
Menzato et al [43]	2022	Guinea-Bissau	Cross sectional Study	898	Adults	0	Sickle scan (POCT)
Oluwole et al [44]	2022	Nigeria	Cross sectional Study	250	Children	15	HPLC
Tutuba et al [45]	2022	Tanzania	Cross sectional Study	300	Adults	1	Sickle scan (POCT)
Akinbodewa et al [46]	2021	Nigeria	Cross sectional Study	102	Adults	0	HB Electrophoresis
Alagbe et al [47]	2021	Nigeria	Retrospective Study	942	Mixed Age groups	67	HB Electrophoresis
Hernandez et al [22]	2021	Uganda	Retrospective Study	278651	Children	7664	IsoElectric Focussing
Ezenwosu et al [48]	2021	Nigeria	Cross sectional Study	10167	Adults	5	HB Electrophoresis
Islam et al [49]	2021	Nigeria	Cross sectional Study	11420	Children	151	Sickle scan (POCT)
Nnodu et al [50]	2021	Nigeria	Retrospective Study	11186	Children	89	Sickle scan
Tegha et al [51]	2021	Malawi	Retrospective Study	10529	New born	14	IsoElectric Focussing
Ahmed et al [52]	2020	Kenya	Cross sectional Study	574	Children	4	HB Electrophoresis
Ambrose et al [53]	2020	Tanzania	Retrospective Study	17200	Children	210	IsoElectric Focussing
Eastburg [54]	2020	Tanzania	Prospective Study	999	New born	30	IsoElectric Focussing
Fenomanana et al [55]	2020	Madagascar	Prospective Study	427	Adults	0	HB Electrophoresis
Kosiyo et al [56]	2020	Kenya	Cross sectional Study	217	Children	24	PCR
Kweka et al [57]	2020	Tanzania	Cross sectional Study	431	Adults	0	PCR
Mohammed-Nafi’u et al [58]	2020	Nigeria	Cross sectional Study	311	New born	6	HPLC
Ngwengi et al [59]	2020	Cameroon	Cross sectional Study	100	Adults	0	HB Electrophoresis
Nnodu et al [12]	2020	Nigeria	Prospective Study	3603	New born	51	Sickle scan (POCT)
Nwabuko et al [1]	2020	Nigeria	Retrospective Study	8457	Mixed Age groups	113	HB Electrophoresis
Oluwole et al [13]	2020	Nigeria	Cross sectional Study	250	Children	1	HemotypeSC™
Oppong et al [60]	2020	Ghana	Cross sectional Study	938	Children	150	IsoElectric Focussing
Wegmuller et al [61]	2020	Ghana	Retrospective Study	1232	Mixed Age groups	10	PCR
Zohoun et al [62]	2020	Benin	Retrospective Study	1483	Adults	0	HB Electrophoresis
Borges et al [63]	2019	Angola	Cross sectional Study	359	New born	12	PCR
Dhabangi et al [64]	2019	Uganda	Case control	196	Children	15	HB Electrophoresis
Kingsley et al [65]	2019	Nigeria	Retrospective Study	3648	Adults	78	HB Electrophoresis
Kiyaga et al [21]	2019	Uganda	Prospective Study	163334	Children	1422	PCR
Kuta et al [66]	2019	Kenya	Cross sectional Study	1810	New born	57	IsoElectric Focussing
Macharia et al [67]	2019	Kenya	Cross sectional Study	15301	New born	122	HPLC
Nankanja et al [68]	2019	Uganda	Prospective Study	1000	Children	96	HB Electrophoresis
Nkya et al [69]	2019	Tanzania	Cross sectional Study	3981	New born	31	IsoElectric Focussing
Uyoga et al [70]	2019	Kenya	Prospective Study	15702	Children	128	HPLC
Watenga et al [71]	2019	Kenya	Cross sectional Study	225	Adults	0	Sickle scan (POCT)
Ada et al [72]	2018	Nigeria	Cross sectional Study	383	Mixed Age groups	10	HB Electrophoresis
Anabire et al [73]	2018	Ghana	Cross sectional Study	150	Children	15	HB Electrophoresis
David et al [74]	2018	Nigeria	Cross sectional Study	208	Children	0	HB Electrophoresis
Dodo et al [75]	2018	Benin	Retrospective Study	813	Mixed Age groups	147	HB Electrophoresis
McGann et al [76]	2018	Malawi	Cross sectional Study	1071	Children	1	HB Electrophoresis
Makani et al [77]	2018	Tanzania	Prospective Study	6397	Mixed Age groups	3751	HPLC
Mpimbaza et al [78]	2018	Uganda	Case control	945	Children	8	HB Electrophoresis
Musyoka et al [79]	2018	Tanzania	Cross sectional Study	50	Children	9	Sodium Metabisulfite
Tossea et al [80]	2018	Côte d’Ivoire	Cross sectional Study	794	Mixed Age groups	5	HB Electrophoresis
Wirth et al [81]	2018	Sierra Leone	Cross sectional Study	643	Mixed Age groups	25	HB Electrophoresis
Englestone et al [82]	2017	Cameroon	Cross sectional Study	291	Children	5	HPLC
Jiya et al [83]	2017	Nigeria	Cross sectional Study	395	Children	44	HB Electrophoresis
Lwanira et al [84]	2017	Uganda	Prospective Study	414	Children	1	PCR
Owusu et al [85]	2017	Ghana	Cross sectional Study	714	Mixed Age groups	7	PCR
Ademuyiwa et al [86]	2016	Nigeria	Prospective Study	124	Children	1	HB Electrophoresis
Adu et al [87]	2016	Ghana	Cross sectional Study	200	Adults	0	HB Electrophoresis
Burnham et al [88]	2016	Nigeria	Prospective Study	3371	Adults	4	HPLC
Maeder et al [89]	2016	Madagascar	Cross sectional Study	807	Children	19	PCR
Moez et al [90]	2016	Egypt	Cross sectional Study	349	Children	6	HB Electrophoresis
Okocha et al [91]	2016	Nigeria	Cross sectional Study	82	Children	0	HB Electrophoresis
Saganuwan et al [92]	2016	Nigeria	Retrospective Study	319	Mixed Age groups	102	HB Electrophoresis
Tubman et al [93]	2016	Liberia	Cross sectional Study	2785	Children	33	HB Electrophoresis
Adeboye et al [94]	2015	Nigeria	Cross sectional Study	167	Children	6	HB Electrophoresis
Lopera-Mesa et al [95]	2015	Mali	Prospective Study	1543	Children	2	HPLC
Adewara et al [96]	2014	Nigeria	Cross sectional Study	1086	Adults	13	HB Electrophoresis
Amoako et al [97]	2014	Ghana	Cross sectional Study	341	Children	3	HB Electrophoresis
Kondani et al [98]	2014	Democratic Republic of Congo	Prospective Study	247	Children	19	IsoElectric Focussing
Loembet et al [99]	2014	Gabon	Cross sectional Study	4250	Adults	0	IsoElectric Focussing
Suchdev et al [100]	2014	Kenya	Cross sectional Study	858	Children	14	PCR
Ademuyiwa et al [101]	2013	Nigeria	Retrospective Study	95	Children	1	HB Electrophoresis
McGann et al [102]	2013	Angola	Prospective Study	36453	New born	550	IsoElectric Focussing
Nnaji et al [103]	2013	Nigeria	Cross sectional Study	424	Adults	4	HB Electrophoresis
Gahutu et al [104]	2012	Rwanda	Cross sectional Study	749	Children	1	HB Electrophoresis
Lussiana et al [105]	2012	Angola	Retrospective Study	1127	Mixed Age groups	403	HB Electrophoresis
Millimono et al [106]	2012	Guinea	Cross sectional Study	187	Adults	0	HPLC
Napon et al [107]	2012	Burkina Faso	Cross sectional Study	74	Adults	0	HB Electrophoresis
Danquah et al [108]	2010	Ghana	Cross sectional Study	2108	Children	7	PCR
Nisreen et al [109]	2010	Sudan	Cross sectional Study	100	Mixed Age groups	20	HB Electrophoresis
Okwi et al [110]	2010	Uganda	Cross sectional Study	656	Children	11	HB Electrophoresis
Ouedraogo et al [111]	2010	Burkina Faso	Retrospective Study	277	Adults	0	HB Electrophoresis
Umoh et al [112]	2010	Nigeria	Retrospective Study	8097	Adults	121	HB Electrophoresis
Agasa et al [113]	2009	Democratic Republic of Congo	Cross sectional Study	520	Children	5	IsoElectric Focussing
Diallo et al [114]	2009	Burkina Faso	Retrospective Study	173	Mixed Age groups	18	HB Electrophoresis
Kafando et al [115]	2009	Burkina Faso	Prospective Study	2341	New born	47	IsoElectric Focussing
Komba et al [116]	2009	Kenya	Retrospective Study	34529	Children	555	HB Electrophoresis
Okwi et al [117]	2009	Uganda	Cross sectional Study	857	Children	11	HB Electrophoresis
Rahimy et al [118]	2009	Benin	Cross sectional Study	719	Adults	16	HB Electrophoresis
Tshilolo [119]	2009	Democratic Republic of Congo	Cross sectional Study	31204	Children	428	IsoElectric Focussing
Odunvbun et al	2008	Benin	Retrospective Study	644	New born	18	IsoElectric Focussing
Kifude et al [120]	2007	Kenya	Cross sectional Study	584	Children	5	HPLC
Nacoulma et al [121]	2007	Burkina Faso	Retrospective Study	83	Mixed Age groups	3	HB Electrophoresis
Simpore et al [122]	2007	Burkina Faso	Cross sectional Study	18383	Children	46	HB Electrophoresis
Jeremiah et al [123]	2006	Nigeria	Cross sectional Study	620	Adults	0	HB Electrophoresis
Masmas et al [124]	2006	Guinea-Bissau	Prospective Study	1057	Children	2	HPLC
Khelili et al [125]	2004	Tunisia	Mixed study design	1514	Mixed Age groups	21	HB Electrophoresis
Moormann et al [126]	2003	Kenya	Cross sectional Study	696	Mixed Age groups	0	PCR
Deyde et al [127]	2002	Mauritania	Cross sectional Study	700	Adults	0	IsoElectric Focussing
Simpore et al [128]	2002	Burkina Faso	Cross sectional Study	10166	Children	196	HB Electrophoresis
Simpore et al [129]	2002	Burkina Faso	Cross sectional Study	9201	Children	12	HB Electrophoresis
Mockenhaupt et al [130]	2000	Ghana	Cross sectional Study	530	Adults	4	HB Electrophoresis
Aluoch [131]	1997	Kenya	Cross sectional Study	728	Children	20	HB Electrophoresis
Esimai et al [132]	1995	Nigeria	Cross sectional Study	152	Children	38	HB Electrophoresis

### Distribution of included studies by country

**Fig 2:**
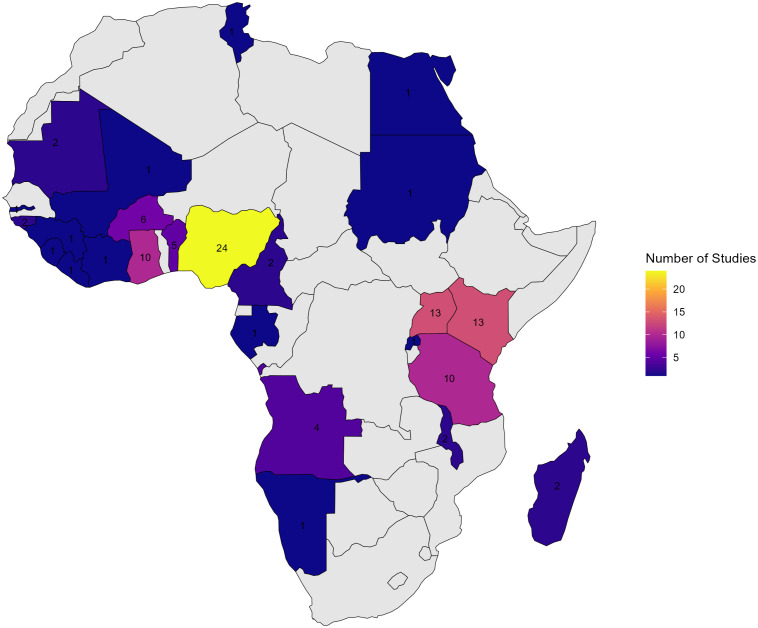
Number of studies included in the meta-analysis by country in Africa. Map created by authors using shapefiles from GADM (Database of Global Administrative Areas, version 4.1, www.gadm.org).

Nigeria contributed the largest number of studies [24], followed by Kenya and Uganda (13 each), Ghana and Tanzania (10 each), Burkina Faso and the Democratic Republic of the Congo (6 each), and Benin [5] (Fig 2). Several other countries contributed one or two studies.

### Prevalence of sickle cell anaemia (HBSS) in Africa

**Fig 3:**
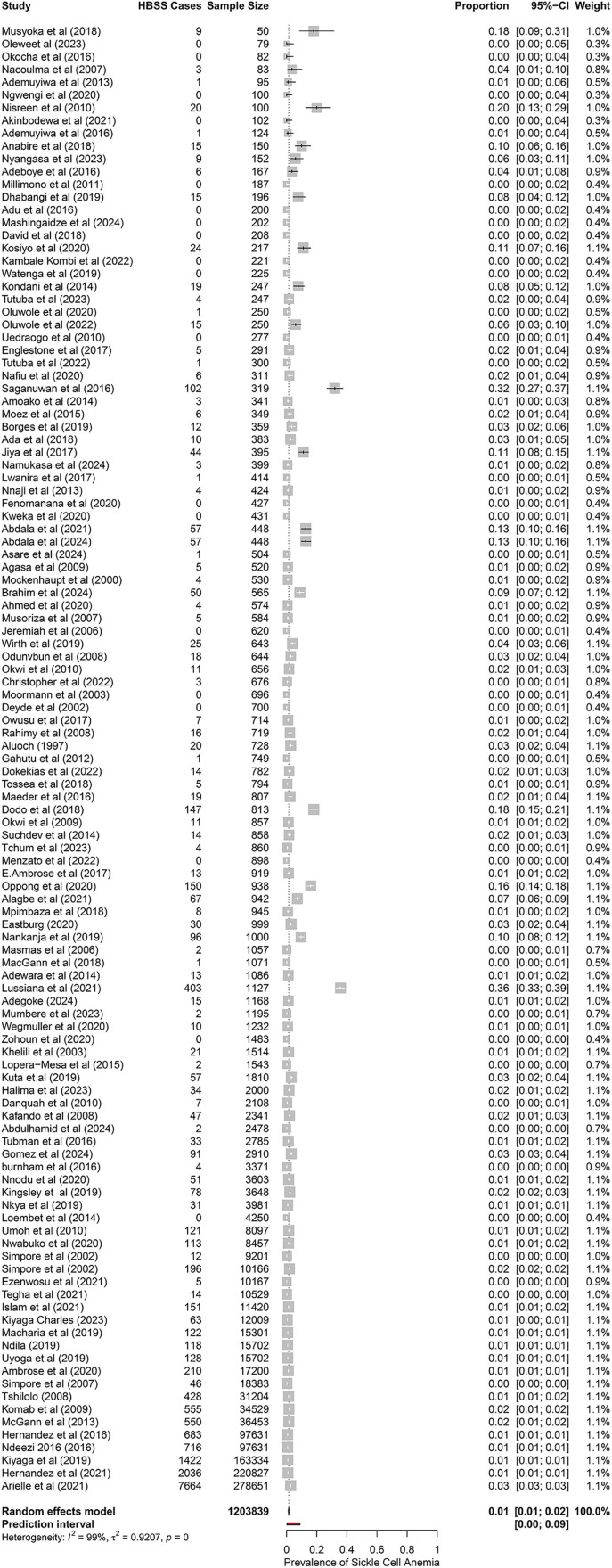
Forest plot showing the pooled prevalence of sickle cell anemia (HBSS) in Africa.

The pooled prevalence of sickle cell anemia (SCA) in Africa due to HbSS hemoglobinopathies was 1.43% (95% CI: 1.08%–1.88%), based on 115 studies, including 1,203,839 participants and 17,458 HbSS cases. Heterogeneity was high (I^2^ = 99.1%, p < 0.001), with prevalence estimates ranging from 0% to 35.76% (Fig 3). The prediction interval was 0.21%–8.91%.

### Regional variation in the prevalence of SCA (HBSS) across Africa

**Fig 4:**
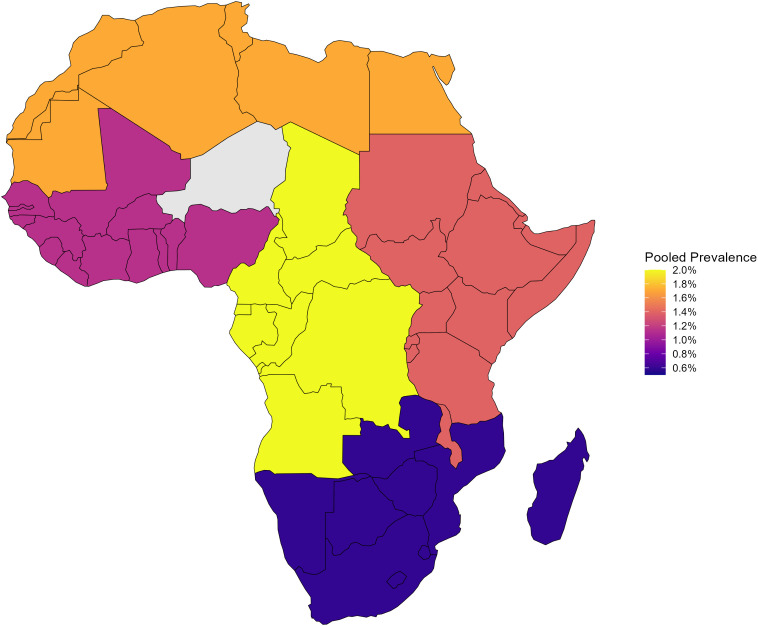
Regional variation in the prevalence of sickle cell anemia (HBSS) across Africa. Map created by authors using shapefiles from GADM (Database of Global Administrative Areas, version 4.1, www.gadm.org).

There was significant heterogeneity in the prevalence of HbSS across African regions (Fig 4). The highest pooled prevalence estimates were observed in Central Africa (1.99%, 95% CI: 0.81%–5.20%, p < 0.001) and North Africa (1.72%, 95% CI: 0.26%–6.64%, p < 0.001). East Africa showed a pooled prevalence of 1.40% (95% CI: 0.91%–2.32%), while West Africa had a prevalence of 1.13% (95% CI: 0.78%–1.77%). The lowest pooled prevalence was recorded in Southern Africa (0.59%, 95% CI: 0.05%–4.78%, p = 0.033). Heterogeneity was high across all regions (I^2^ > 95%), except in Southern Africa (I^2^ = 70.6%). West Africa contributed the largest number of studies (n = 53), whereas Southern Africa was underrepresented with only three studies.

### Effect of study designs

There was substantial variation in the prevalence of sickle cell anemia across the study designs (I^2^ > 96%). The pooled prevalence was 1.22% (95% CI: 0.89%–1.64%) among cross-sectional studies (n = 72), 1.92% (95% CI: 1.20%–3.07%) among retrospective studies (n = 40), and 1.40% (95% CI: 0.98%–2.00%) among prospective studies (n = 2). Two case–control studies and one quasi-experimental study were also included, with a limited contribution to the pooled estimates. Meta-regression indicated that study design did not significantly explain heterogeneity (QM = 4.10, p = 0.663).

### Methods used to diagnose SCA across studies in Africa

Electrophoretic techniques, specifically hemoglobin electrophoresis and isoelectric focusing, have been the most commonly used methods for diagnosing HbSS. The pooled prevalence among studies using hemoglobin electrophoresis (n = 52) was 1.58% (95% CI: 1.01%–2.48%, I^2^ = 98.9%), while for isoelectric focusing (n = 21), it was 1.35% (95% CI: 0.95%–1.91%, I^2^ = 99.6%). Among studies using chromatographic methods (HPLC; n = 13), the pooled prevalence was 1.10% (95% CI: 0.65%–1.84%, I^2^ = 94.7). The molecular method (PCR; n = 15) showed a pooled prevalence of 0.97% (95% CI: 0.62%–1.51%, I^2 ^= 93.7%). Rapid sickling tests (POCT), including HemotypeSC™ and Sickle scan (n = 6), had a pooled prevalence of 1.26% (95% CI: 0.92%–1.71%, I^2^ = 44.4%). Only one study used the biochemical method (sodium metabisulfite), conducted among anemic children at Mbeya Referral Hospital, Southern Tanzania (n = 50), which reported a prevalence of 18% (9/50) [79]. Meta-regression using Diagnostic Method as a moderator indicated that the type of diagnostic method significantly explained some of the variability in prevalence across studies (QM(df = 8) = 16.73, p = 0.0331), although substantial residual heterogeneity remained (tau^2^ = 0.944, I^2^ = 98.9%).

### Temporal variation of SCA (HBSS) in Africa over the last 30 years (1994–2024)

**Fig 5:**
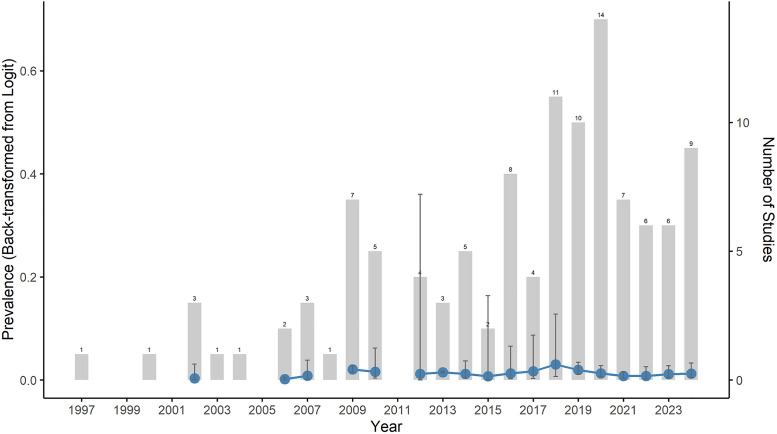
Temporal trends in sickle cell anemia prevalence across Africa. (blue line, left axis) back-transformed from the logarithmic scale and annual research output (grey bars, right axis) from 1994 to 2024. Numbers above the gray bars indicate the number of accessed studies published each year.

The pooled prevalence of Sickle Cell Anemia (SCA) due to the hemoglobin variant HBSS has remained low and relatively stable across studies conducted in Africa between 1994 and 2024 (Fig 5). Annual pooled prevalence estimates were available from 2002 to 2024 and generally ranged between 0.1% and 3%, with most estimates falling between 1% and 2%. The number of studies increased from 1–3 per year between 1997 and 2003 to a peak of 14 in 2020, followed by a steady output of 6–9 studies annually thereafter. Despite the increase in research activity, annual pooled prevalence estimates showed overlapping 95% confidence intervals across years (ranging from approximately 0.03%–0.07% in the lower bounds to 2.8%–7.3% in the upper bounds), indicating no significant temporal change. This was further supported by meta-regression analysis including 115 studies, which showed no statistically significant association between the study year and prevalence (QM(22) = 20.82, p = 0.53). Residual heterogeneity remained high (tau^2^ = 1.21, I^2 ^= 98.9%). The wide prediction interval (0.04%–28.48%) reflects the considerable between-study variability in the reported prevalence.

### Age and sex variation

Sex-specific analyses showed a pooled prevalence of HBSS in males and females, with high heterogeneity across studies. The test for subgroup differences between the sexes yielded Cochran’s Q statistic Q(1) = 0.15, p = 0.694. Age-stratified analyses also showed substantial heterogeneity across children, adults, newborns, and mixed-age populations, with all subgroup heterogeneity tests being statistically significant (p < 0.001 for each) (Table 2).

**Table 2:** Pooled prevalence of sickle cell anemia (HBSS) by Sex and Age in Africa.

Subgroup	Number of studies	Pooled prevalence (%)	95% CI (%)	I² (%)
**Sex**				
Males	22	2.71	1.33–5.44	95.9
Females	28	1.74	0.90–3.34	96.8
**Age category**				
Children	53	1.65	1.29–2.11	99.1
Adults	27	0.45	0.26–0.76	91.8
Mixed-age	17	3.24	1.45–7.09	99.0
Newborns	18	1.28	1.00–1.64	95.3

### Regression analysis

Meta-regression analyses showed that region, study design, and sample size did not significantly explain the heterogeneity (p > 0.19). Diagnostic method was a significant moderator (χ² = 16.73, df = 8, p = 0.033), and age category also influenced the HBSS prevalence (χ² = 33.66, df = 3, p < 0.0001). In the multivariate model, including all moderators, the overall effect was marginally significant (χ² = 29.01, df = 19, p = 0.066), accounting for some variability; however, substantial residual heterogeneity persisted (tau^²^ = 1.014, I^2^ = 98.6%).

### Sensitivity analysis: Leave-one-out approach

The leave-one-out sensitivity analysis revealed that no single study had a significant impact on the overall prevalence estimate of HBSS (S5 Table). Throughout the iterations, the prevalence remained consistent, with minimal absolute differences ranging from 0.0000 to 0.0017 and percentage changes between 0.00% and 2.82%. The exclusion of Makani et al. (2021) [22] resulted in the largest shift, causing a 2.84% change in the pooled prevalence estimate. The original pooled prevalence was 0.0130 (95% CI: 0.0094–0.0181), and the removal of individual studies produced estimates between 0.0130 and 0.0147. The median absolute difference was 0.0001, with a median percentage change of 0.38%.

### Assessment of publication bias

**Fig 6:**
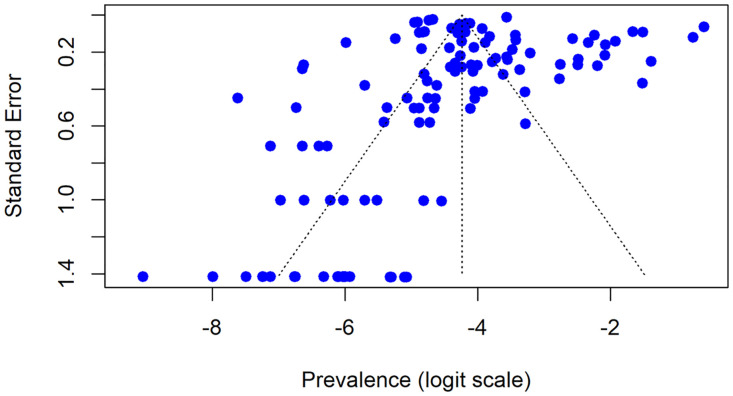
Funnel plot.

Egger’s linear regression test did not indicate statistically significant evidence of publication bias (t = –0.32, df = 113, p = 0.752) (Fig 6). The bias estimate was –0.36 with a standard error of 1.13. The analysis showed a τ² value of 107.73, confirming substantial residual heterogeneity across studies.

### Sources of heterogeneity

Regression analyses exploring potential sources of heterogeneity among studies showed that age category was a significant driver of the observed variation in the prevalence of HBSS ((χ²(df = 3) = 33.659, *p* < 0.0001).

### Certainty of evidence

The methodological quality of the pooled estimates was assessed using the AMSTAR 2 tool. The review met 16 of the 17 AMSTAR 2 criteria, indicating a high methodological confidence (S1 Fig). However, the substantial heterogeneity across studies, especially in diagnostic methods and age groups, may limit the generalizability of the estimates.

## Discussion

This systematic review and meta-analysis provides a comprehensive synthesis of sickle cell anemia (SCA) prevalence due to HbSS across Africa, analyzing 115 studies encompassing nearly 1.2 million participants. We estimated a pooled prevalence of 1.43% (95% CI: 1.08%–1.88%), though this summary estimate must be interpreted cautiously given the extreme heterogeneity (I^2^ = 99.1%) and wide prediction interval (0.21%–8.91%), indicating substantial variation across populations and settings.

The extraordinarily high heterogeneity reflects the genuine biological, geographical, and methodological diversity. Regional prevalence differences, from 2.0% in Central Africa to 0.6% in Southern Africa, align with evolutionary biology linking HbS allele frequency to historical malaria endemicity [133]. Regions with sustained malaria transmission exert stronger selective pressure favoring the protective HbAS heterozygous state, indirectly maintaining higher HbSS frequencies through Hardy-Weinberg dynamics [134–136]. Central Africa’s elevated prevalence supports the hypothesis that this region represents the origin point of the sickle mutation approximately 5,000–10,000 years ago [137,138], with subsequent migrations distributing the allele continentally.

Beyond geographic variation, sampling frame differences critically influence the estimates. Hospital-based convenience samples may overestimate the prevalence by enriching symptomatic individuals, while community newborn screening provides unbiased population estimates. Urban tertiary centers capture different demographics than rural ones. Unfortunately, inconsistent reporting prevented formal subgroup analysis by sampling frame, a significant limitation that requires future attention. The markedly lower adult prevalence (0.45%) compared to that in children (1.65%) and newborns (1.28%) provides compelling evidence of high early mortality. With 50–90% of affected children dying before the age of five in resource-limited settings [18,19], cross-sectional adult studies systematically underestimate birth prevalence. This survival bias explains why newborn screening provides the most accurate population-level estimate.

Although meta-regression identified the diagnostic method as a significant moderator (χ² = 16.73, p = 0.033), misclassification concerns remained. Electrophoretic techniques confirmed 86.4% of HbSS cases but vary in specificity. Standard alkaline hemoglobin electrophoresis reliably distinguishes HbSS from HbAA and HbAS, yet may not differentiate HbSS from rarer variants (HbSD, HbSO-Arab) without confirmatory testing [139]. Only 15 studies (13%) employed molecular methods representing the gold standard. One study using solely sodium metabisulfite testing [79], which cannot distinguish HbSS from HbAS, poses overestimation risk, though its small sample (n = 50) minimally impacted pooled estimates. However, subtle misclassification may persist in studies lacking confirmatory testing, particularly where compound heterozygotes are prevalent.

The statistical stability of pooled estimates across three decades (approximately 1.4–1.5% from 1994–2024) requires careful interpretation. While annual estimates showed overlapping confidence intervals, the wide prediction interval indicates substantial individual study variation. This paradox reflects that early epidemiological studies successfully captured broad continental patterns despite limited diagnostic capabilities, and meta-regression confirmed no significant temporal trend (QM = 20.82, p = 0.53). Yet persistent high heterogeneity shows that pooled estimate stability masks geographic and methodological variation. Six-fold increased research output (from 1–3 studies pre-2003–14 in 2020) expanded coverage but did not shift overall estimates, suggesting increased sampling reinforced rather than revised our understanding while revealing greater local variability. Critically, temporal stability should not suggest unchanging disease burden within populations. Birth prevalence may remain stable while mortality, severity, and life expectancy change substantially with improved healthcare access.

Southern Africa’s severe underrepresentation (only 3 studies) critically limits generalizability. This region exhibits distinct population genetics, lower historical malaria endemicity, and different demographic histories than West and Central Africa. The 0.59% estimate rests on insufficient evidence and warrants extreme caution. North Africa similarly contributed limited data (n = 3), and several nations had no representation. This geographic bias likely reflects publication patterns and research infrastructure concentration rather than SCA absence. Future research must prioritize underrepresented regions and systematically search African-specific databases beyond international platforms.

Egger’s test suggested no significant publication bias (t = –0.32, p = 0.752), though with high heterogeneity. Traditional assessments assume relatively homogeneous underlying effects, whereas our analysis encompasses genuinely diverse populations. The non-significant result may indicate that wide prevalence ranges (0%–35.76%) reflect authentic diversity rather than selective reporting. Nevertheless, subtle biases may persist, where studies reporting unexpectedly low prevalence may face publication barriers, while high-prevalence endemic-area studies receive preferential reporting. Language bias concerns persist given our English-language database focus, potentially missing French, Portuguese, or Arabic publications.

These findings carry actionable policy implications. Two- to three-fold regional variation necessitates tailored screening strategies rather than uniform continental policies, where high-burden regions require universal newborn screening while lower-prevalence areas might adopt risk-based targeting. Persistent electrophoretic method dominance (86.4% of diagnoses) suggests delayed point-of-care technology adoption that could expand rural screening access, though quality assurance must ensure accuracy. Absent temporal trends may reflect inadequate surveillance rather than stable burden, thus population-based registries with standardized protocols would enable meaningful epidemiological shift detection. Most critically, the observed childhood-to-adulthood prevalence decline underscores urgent need for comprehensive care programs addressing 50–90% under-five mortality. Evidence-based interventions including penicillin prophylaxis, pneumococcal vaccination, hydroxyurea therapy, and parent education demonstrate effectiveness but remain underutilized across Africa.

## Limitations

Beyond acknowledged geographic underrepresentation and high heterogeneity, several limitations merit emphasis. The sensitivity analysis influence threshold (0.002 absolute prevalence difference) was pragmatically chosen but requires validation using alternative metrics (DFBETAS, Cook’s distance). Inconsistent reporting of urban/rural settings, facility levels, and sampling procedures prevented granular subgroup analyses better explaining heterogeneity. Lack of systematic African-specific database screening (AJOL, WHO African Index Medicus) represents a gap requiring future correction. Pre-specified analyses stratifying by sampling frame (community versus clinical), screening versus convenience samples, and malaria endemicity zones would provide more robust heterogeneity exploration. Finally, inaccessible full texts for 15 potentially eligible studies may introduce selection bias of unclear direction and magnitude.

## Conclusion

SCA represents a substantial but geographically heterogeneous public health challenge across Africa, with 1.43% pooled prevalence masking variation from <1% to >3% across regions. Extreme heterogeneity reflects authentic diversity in genetic epidemiology, population structure, survival patterns, and methodological approaches rather than statistical artifact. Findings mandate region-specific interventions, expanded diagnostic access with quality assurance, systematic surveillance infrastructure, and continued research addressing geographic and methodological gaps. Future work must prioritize underrepresented regions, standardize diagnostic protocols, and establish prospective cohorts tracking mortality and morbidity trends alongside prevalence patterns.

### Patient and public involvement statement

Patients or the public were not involved in the design, conduct, reporting, or dissemination of our research.

### Consent to participate

Not applicable.

### Consent for publication

Not applicable.

### Reporting guideline compliance

This review was conducted in accordance with the PRISMA 2020 guidelines.

## Supporting information

S1 Table Preferred Reporting Items for Systematic Reviews and Meta-Analyses (PRISMA) checklist. (PDF)

S2 Table Search tables for PubMed, Google scholar, Scopus and BASE databases from 1994–2024. (PDF)

S3 Table Studies excluded with reasons for exclusion. (PDF)

S4 Table Risk of bias analysis using JBI critical appraisal tool for prevalence studies. (PDF)

S5 Table Results of the Leave-Out-One sensitivity analysis. (PDF)

S1 Fig Certainty of evidence assessment using A Measurement Tool to Assess systematic Reviews (AMSTAR). (PDF)

## Abbreviations

SCA: sickle cell anemia
SCD: sickle cell disease
HBSS: hemoglobin SS (homozygous sickle cell genotype)
HbS: hemoglobin S
HbF: fetal hemoglobin
β⁰: beta-zero thalassemia (absence of beta-globin production)
β⁺: beta-plus thalassemia (reduced beta-globin production)
HPLC: high-performance liquid chromatography
PCR: polymerase chain reaction
POCT: point-of-care testing
