# Supplementary material for: Burden of sickle cell anemia in Africa: A systematic review and meta-analysis
Source: PLoS One. 2025 Nov 25;20(11):e0337090. doi: 10.1371/journal.pone.0337090 (PMC12646443; doi:10.1371/journal.pone.0337090)
Supplement: S3 Table — (PDF) [file pone.0337090.s003.pdf]

S2 Table. studies that appeared to meet the inclusion criteria, but which were excluded, and explain why they were excluded

Title	Year	Reason for exclusion
Correlates of steady-state haematocrit and hepatosplenomegaly in children with sickle cell disease in Western Nigeria.	2012	High Risk of Bias
Epidemiological and molecular study of hemoglobinopathies in Mauritanian patients.	2022	High Risk of Bias
Prevalence and Predictive Factors of Sickle Cell Emergencies Readmission in the Clinical Hematology Department of Dakar, Senegal.	2024	High Risk of Bias
Renal Abnormalities among Sickle Cell Disease Patients in a Poor Management Setting: A Survey in the Democratic Republic of the Congo.	2022	High Risk of Bias
Sickle cell disease in Sierra Leone: a neglected problem	2015	High Risk of Bias
Sickle cell disease in the Zanzibar Archipelago, the Republic of Tanzania.	2024	High Risk of Bias
Simultaneous point-of-care detection of anemia and sickle cell disease in Tanzania: the RAPID study.	2018	High Risk of Bias
Stroke in children in Yaounde, Cameroon.	1994	High Risk of Bias
The burden of sickle cell disease in Cape Town.	2012	High Risk of Bias
The prevalence of sickle cell trait in Sierra Leone. A laboratory profile.	1996	High Risk of Bias
[Pediatric management of sickle cell disease: experience at the Charles de Gaulle University Children's Hospital in Ouagadougou (Burkina Faso)].	2008	Insufficient or Unclear Diagnostic Method
[Seroprevalence of human parvovirus B19 in children with fever and rash in the North of Tunisia].	2016	Insufficient or Unclear Diagnostic Method
Post-hospital mortality in children aged 2-12 years in Tanzania: A prospective cohort study.	2018	Insufficient or Unclear Diagnostic Method
Prevalence and pattern of sickle cell disease among children attending tertiary and non-tertiary health care institution in South-Eastern State, Nigeria: A 10 …	2016	Insufficient or Unclear Diagnostic Method
Prevalence and predictors of hypoxaemia in respiratory and non-respiratory primary diagnoses among emergently ill children at a tertiary hospital in south western Nigeria.	2013	Insufficient or Unclear Diagnostic Method
Prevalence, determinants and impact of haemoglobin phenotype misdiagnosis among parents of children living with sickle cell disease in Nigeria	2021	Insufficient or Unclear Diagnostic Method
Trends and causes of maternal death at the Lagos University teaching hospital, Lagos, Nigeria (2007-2019).	2022	Insufficient or Unclear Diagnostic Method
A CROSS-SECTIONAL STUDY OF THE PREVALENCE OF SICKLE CELL DISEASE AMONG CHILDREN OF UNDER THE AGE OF FIVE YEARS AT HERI MISSION HOSPITAL IN BUHIGWE DISTRICT – KIGOMA, TANZANIA	2023	Not a Sickle Cell Prevalence Study
A Groundbreaking Prevalence Survey Conducted in Mauritania for Enhanced Sickle Cell Control	2024	Not a Sickle Cell Prevalence Study

A Study of the Prevalence and Pattern of Sickle Cell Retinopathy among Eye Clinic Attendees in a Nigerian Tertiary Hospital.	2023	Not a Sickle Cell Prevalence Study
Acceptabilité du dépistage néonatal de la drépanocytose au cours de la pandémie au COVID-19 à Kisangani, en République Démocratique du Congo ; Neonatal screening to identify infants with sickle cell disease during the Covid-19 pandemic in Kisangani, Democratic Republic of Congo	2020	Not a Sickle Cell Prevalence Study
Adapting the preterm birth phenotyping framework to a low-resource, rural setting and applying it to births from Migori County in western Kenya.	2023	Not a Sickle Cell Prevalence Study
Association between haemoglobin variants S and C and Mycobacterium ulcerans disease (Buruli ulcer): a case-control study in Benin.	2007	Not a Sickle Cell Prevalence Study
Avascular necrosis of the femoral head in sickle cell disease in Nigeria: a retrospective study.	2007	Not a Sickle Cell Prevalence Study
Diagnosis patterns of sickle cell disease in Ghana: a secondary analysis.	2021	Not a Sickle Cell Prevalence Study
DNA testing for sickle cell anemia in Africa: Implementation choices for the Democratic Republic of Congo.	2022	Not a Sickle Cell Prevalence Study
Efficacy of premarital genotype screening and counselling on knowledge and attitude toward sickle cell disease among university students in Dodoma Tanzania: uncontrolled quasi-experimental study.	2020	Not a Sickle Cell Prevalence Study
Emergency Blood Transfusion in Children in a Tertiary Hospital in Nigeria: Indications, Frequency and Outcome.	2018	Not a Sickle Cell Prevalence Study
Epidemiology, clinical presentation, and treatment of sickle cell disease: A retrospective study in Wangata General Referral Hospital, Mbandaka, Democratic Republic of Congo	2024	Not a Sickle Cell Prevalence Study
Evaluation of sociodemographic, clinical, and laboratory markers of sickle leg ulcers among young nigerians at a tertiary health institution.	2018	Not a Sickle Cell Prevalence Study
Haemoglobin F, A2, and S levels in subjects with or without sickle cell trait in south-eastern Gabon.	2017	Not a Sickle Cell Prevalence Study
Hemoglobin variants identified in the Uganda Sickle Surveillance Study.	2016	Not a Sickle Cell Prevalence Study
Hyperglycemia in Acutely Ill Non-diabetic Children in the Emergency Rooms of 2 Tertiary Hospitals in Lagos, Nigeria.	2016	Not a Sickle Cell Prevalence Study
Impact and burden of sickle cell disease in critically ill obstetric patients in a high dependency unit in Sierra Leone—a registry based evaluation	2023	Not a Sickle Cell Prevalence Study
Ischemic priapism in South-East Nigeria: Presentation, management challenges, and aftermath issues.	2016	Not a Sickle Cell Prevalence Study
Maternal and fetal outcomes of jaundice in pregnancy at the University College Hospital, Ibadan.	2009	Not a Sickle Cell Prevalence Study
Maternal mortality in Ghana: a hospital-based review.	2012	Not a Sickle Cell Prevalence Study
Operational analysis of the national sickle cell screening programme in the Republic of Uganda.	2021	Not a Sickle Cell Prevalence Study

Paediatric sickle cell disease at a tertiary hospital in Malawi: a retrospective cross-sectional study.	2021	Not a Sickle Cell Prevalence Study
Paper-based microchip electrophoresis for point-of-care hemoglobin testing.	2020	Not a Sickle Cell Prevalence Study
Prevalence of Association of Glucose-6-Phosphate Dehydrogenase Deficiency and Sickle Cell Disease at the National Teaching Hospital of Cotonou in Benin	2023	Not a Sickle Cell Prevalence Study
Prevalence of sickle cell disease among children attending plateau specialist hospital, Jos, Nigeria	2018	Resistry based satudy/No primary data was used
[Incidence of sickle cell disease in Equatorial Guinea].	2011	Used Only Confirmed Cases / Secondary Data
Prevalence of sickle cell disease among hospitalised children and caregivers' knowledge on risk factors associated with sickle cell crises in Kigoma Region, Tanzania	2021	Used Only Confirmed Cases / Secondary Data
Prevalence of sickle cell disease among pregnant women in a tertiary health center in south-south Nigeria	2016	Used Only Confirmed Cases / Secondary Data
Prevalence of sickle cell traits in samples from Western Uganda and validation of one spot restriction fragment length polymorphism assay at the Central Public Health …	2023	Used Only Confirmed Cases / Secondary Data
Prevalence of β-S Globin Haplotypes in Jazan Region of Saudi Arabia.	2021	Used Only Confirmed Cases / Secondary Data
Prevalence, Pattern of Disease and Outcome of Children with Sickle Cell Disease Admitted in a Private Health Facility in Southern Nigeria	2023	Used Only Confirmed Cases / Secondary Data
Prevention of hemoglobinopathies in Egypt.	2009	Used Only Confirmed Cases / Secondary Data
Severe anemia in pregnancy in rural Ghana: a case-control study of causes and management.	2006	Used Only Confirmed Cases / Secondary Data
Sickle cell disease clinical phenotypes in Nigeria: A preliminary analysis of the Sickle Pan Africa Research Consortium Nigeria database.	2020	Used Only Confirmed Cases / Secondary Data
Sickle cell disease in anaemic children in a Sierra Leonean district hospital: a case series.	2019	Used Only Confirmed Cases / Secondary Data
Sickle Cell Disease in Mauritania: epidemiological, clinical and therapeutic aspects about 135 cases.	2022	Used Only Confirmed Cases / Secondary Data
Sickle cell disease in pregnancy: trend and pregnancy outcomes at a tertiary hospital in Tanzania.	2013	Used Only Confirmed Cases / Secondary Data
SS Sickle Cell Disease and Severe Malaria in Children Aged 0 to 15 Hospitalized in the Pediatric Department of the Donka CHU National Hospital in Conakry, Republic of Guinea	2023	Used Only Confirmed Cases / Secondary Data
The burden and outcomes of stroke in young adults at a tertiary hospital in Tanzania: a comparison with older adults.	2020	Used Only Confirmed Cases / Secondary Data
