# Supplementary material for: Burden of sickle cell anemia in Africa: A systematic review and meta-analysis
Source: PLoS One. 2025 Nov 25;20(11):e0337090. doi: 10.1371/journal.pone.0337090 (PMC12646443; doi:10.1371/journal.pone.0337090)
Supplement: S4 Table — (PDF) [file pone.0337090.s004.pdf]

Title	Year	Was the sample frame appropriate to address the target populati	Were study participants sampled in an appropriate way?	Was the sample size adequate?	Were the study subjects and the setting described in detail?	Was the data analysis conducted with sufficient coverage of the	Were valid methods used for the identification of the condition	Was the condition measured in a standard, reliable way for all	Was there appropriate statistical analysis?	Was the response rate adequate, and if not, was the low respons	Risk of Bias	Decision
[Chronic osteomyelitis at the Ouagadougou teaching hospital (Burkina Faso). A retrospective study of 102 cases (1996-2000)].	2007	Yes	Yes	Yes	Yes	Yes	Yes	Yes	Yes	Not Applicable	Low	Include
[Emergency treatment of sickle cell diseases in the Blood Diseases Department at the Koutoukou Maga National Teaching Hospital, Cotonou, Benin].	2018	Yes	Yes	Yes	Yes	Yes	Yes	Yes	Yes	Not Applicable	Low	Include
[Prevalence of sickle cell trait in blood donors at the regional blood transfusion center in the Haute Matsiatra Region, Madagascar].	2020	Yes	Yes	Yes	Yes	Yes	Yes	Yes	Yes	Yes	Low	Include
< The> prevalence of sickle cell anaemia in northern areas of algadaref state, Sudan	2010	Yes	Yes	Yes	Yes	Yes	Yes	Yes	Yes	Yes	Low	Include
A prospective newborn screening and treatment program for sickle cell anemia in Luanda, Angola.	2013	Yes	Yes	Yes	Yes	Yes	Yes	Yes	Yes	Yes	Low	Include

A ten year review of the sickle cell program in Muhimbili National Hospital, Tanzania.	2018	Yes	Yes	Yes	Yes	Yes	Yes	Yes	Yes	Yes	Low	Include
Abnormal haemoglobin variants, ABO and Rh blood groups among student of African descent in Port Harcourt, Nigeria.	2006	Yes	Yes	Yes	Yes	Yes	Yes	Yes	Yes	Yes	Low	Include
An overview of sickle cell disease from the socio-demographic triangle - a Nigerian single-institution retrospective study.	2022	Yes	Yes	Yes	Yes	Yes	Yes	Yes	Yes	Not Applicable	Low	Include
An up-date on the prevalence of sickle cell trait in Eastern and Western Uganda.	2010	Yes	Yes	Yes	Yes	Yes	Yes	Yes	Yes	Yes	Low	Include
Anaemia in pregnant Ghanaian women: importance of malaria, iron deficiency, and haemoglobinopathies.	2000	Yes	Yes	Yes	Yes	Yes	Yes	Yes	Yes	Yes	Low	Include
Anemia, micronutrient deficiencies, malaria, hemoglobinopathies and malnutrition in young children and non-pregnant women in Ghana: Findings from a national survey.	2020	Yes	Yes	Yes	Yes	Yes	Yes	Yes	Yes	Yes	Low	Include
Association between haematological parameters and sickle cell genotypes in children with	2020	Yes	Yes	Yes	Yes	Yes	Yes	Yes	Yes	Yes	Low	Include

Plasmodium falciparum malaria resident in Kisumu County in Western Kenya.												
Association between sickle cell and β-thalassemia genes and hemoglobin concentration and anemia in children and non-pregnant women in Sierra Leone: ancillary analysis of data from Sierra Leone's 2013 National Micronutrient Survey.	2018	Yes	Yes	Yes	Yes	Yes	Yes	Yes	Yes	Yes	Low	Include
Association of sickle cell disease with anthropometric indices among under-five children: evidence from 2018 Nigeria Demographic and Health Survey.	2021	Yes	Yes	Yes	Yes	Yes	Yes	Yes	Yes	Yes	Low	Include
Associations between erythrocyte polymorphisms and risks of uncomplicated and severe malaria in Ugandan children: A case control study.	2018	Yes	Yes	Yes	Yes	Yes	Yes	Yes	Yes	Yes	Low	Include
Associations between red cell polymorphisms and Plasmodium falciparum infection in the middle belt of Ghana.	2014	Yes	Yes	Yes	Yes	Yes	Yes	Yes	Yes	Yes	Low	Include
Biological and clinical presentation of patients with	2002	Yes	Yes	Yes	Yes	Yes	Yes	Yes	Yes	Yes	Low	Include

hemoglobinopathies attending an urban hospital in Ouagadougou: confirmation of the modification of the balance between Hb S and Hb C in Burkina Faso.												
Child mortality from sickle cell disease in Nigeria: a model-estimated, population-level analysis of data from the 2018 Demographic and Health Survey.	2021	Yes	Yes	Yes	Yes	Yes	Yes	Yes	Yes	Yes	Low	Include
Clinical and molecular aspects of haemoglobinopathies in Tunisia.	2004	Yes	Yes	Yes	Yes	Yes	Yes	Yes	Yes	Not Applicable	Low	Include
Community based screening for sickle haemoglobin among pregnant women in Benue State, Nigeria: I-Care-to-Know, a Healthy Beginning Initiative.	2021	Yes	Yes	Yes	Yes	Yes	Yes	Yes	Yes	Yes	Low	Include
Comorbidity of sickle cell trait and albinism: a cross-sectional survey in the Democratic Republic of the Congo.	2020	Yes	Yes	Yes	Yes	Yes	Yes	Yes	Yes	Yes	Low	Include
Contribution of malaria and sickle cell disease to anaemia among children aged 6-59 months in Nigeria: a cross-sectional study using data from the	2022	Yes	Yes	Yes	Yes	Yes	Yes	Yes	Yes	Yes	Low	Include

2018 Demographic and Health Survey.												
Cross sectional study on prevalence of sickle cell alleles S and C among patients with mild malaria in Ivory Coast.	2018	Yes	Yes	Yes	Yes	Yes	Yes	Yes	Yes	Yes	Low	Include
Determination of birth prevalence of sickle cell disease using point of care test HemotypeSC™ at Rundu Hospital, Namibia.	2024	Yes	Yes	Yes	Yes	Yes	Yes	Yes	Yes	Not Applicable	Low	Include
Distribution of haemoglobin genotypes, knowledge, attitude and practices towards sickle cell disease among unmarried youths in the Buea Health District, Cameroon.	2020	Yes	Yes	Yes	Yes	Yes	Yes	Yes	Yes	Yes	Low	Include
Early diagnosis of sickle cell disease at birth hospitals and vaccination centers in Angola using point-of-care tests.	2023	Yes	Yes	Yes	Yes	Yes	Yes	Yes	Yes	Yes	Low	Include
Effect of iron fortification on anaemia and risk of malaria among Ghanaian pre-school children with haemoglobinopathies and different ABO blood groups.	2023	Yes	Yes	Yes	Yes	Yes	Yes	Yes	Yes	Yes	Low	Include
Effect of red blood cell variants on childhood malaria in	2015	Yes	Yes	Yes	Yes	Yes	Yes	Yes	Yes	Yes	Low	Include

Mali: a prospective cohort study.												
Epidemiologic profile of hemoglobinopathies in Benin.	2024	Yes	Yes	Yes	Yes	Yes	Yes	Yes	Yes	Not Applicable	Low	Include
Evaluation of hematological indices of childhood illnesses in Tamale Metropolis of Ghana.	2018	Yes	Yes	Yes	Yes	Yes	Yes	Yes	Yes	Yes	Low	Include
Extremely high birth prevalence of sickle cell disease in rural Tanzania.	2020	Yes	Yes	Yes	Yes	Yes	Yes	Yes	Yes	Yes	Low	Include
Feasibility and acceptability of early infant screening for sickle cell disease in Lagos, Nigeria-A pilot study.	2020	Yes	Yes	Yes	Yes	Yes	Yes	Yes	Yes	Yes	Low	Include
Febrile Convulsion among Hospitalized Children Aged Six Months to Five Years and Its Association With Haemoglobin Electrophoretic Pattern.	2015	Yes	Yes	Yes	Yes	Yes	Yes	Yes	Yes	Yes	Low	Include
Frequencies of sickle cell trait and glucose-6-phosphate dehydrogenase deficiency differ in highland and nearby lowland malaria-endemic areas of Kenya.	2003	Yes	Yes	Yes	Yes	Yes	Yes	Yes	Yes	Yes	Low	Include
Genotyping the BCL11A Single Nucleotide Polymorphism and Associated Levels of Fetal Hemoglobin in	2024	Yes	Yes	Yes	Yes	Yes	Yes	Yes	Yes	Yes	Low	Include

Mauritanian Sickle Cell Patients.											
Glucose-6-phosphate dehydrogenase deficiency and sickle cell disease in Burkina Faso.	2007	Yes	Yes	Yes	Yes	Yes	Yes	Yes	Yes	Yes	Low	Include
Haemoglobin genotypes: a prevalence study and implications for reproductive health in Uyo, Nigeria.	2010	Yes	Yes	Yes	Yes	Yes	Yes	Yes	Yes	Not Applicable	Low	Include
HemoTypeSC Demonstrates >99% Field Accuracy in a Sickle Cell Disease Screening Initiative in Children of Southeastern Uganda.	2019	Yes	Yes	Yes	Yes	Yes	Yes	Yes	Yes	Yes	Low	Include
High prevalence of haemoglobin S in the closed Egyptian community of Siwa Oasis.	2016	Yes	Yes	Yes	Yes	Yes	Yes	Yes	Yes	Yes	Low	Include
High prevalence of hemoglobin disorders and glucose-6-phosphate dehydrogenase (G6PD) deficiency in the Republic of Guinea (West Africa).	2012	Yes	Yes	Yes	Yes	Yes	Yes	Yes	Yes	Yes	Low	Include
Higher resistance to Plasmodium falciparum infection in patients with homozygous sickle cell disease in western Kenya.	1997	Yes	Yes	Yes	Yes	Yes	Yes	Yes	Yes	Yes	Low	Include
Hospital Prevalence of Sickle Cell Disease in Children	2024	Yes	Yes	Yes	Yes	Yes	Yes	Yes	Yes	Yes	Low	Include

Under 5 Years of Age in a Region of the Democratic Republic of Congo												
Immune responses to P falciparum antibodies in symptomatic malaria patients with variant hemoglobin genotypes in Ghana.	2024	Yes	Yes	Yes	Yes	Yes	Yes	Yes	Yes	Yes	Low	Include
Implementing newborn screening for sickle cell disease as part of immunisation programmes in Nigeria: a feasibility study.	2020	Yes	Yes	Yes	Yes	Yes	Yes	Yes	Yes	Yes	Low	Include
Incidence of bacteraemia among children with severe anaemia in Wesley Guild Hospital, Ilesa, Nigeria.	1995	Yes	Yes	Yes	Yes	Yes	Yes	Yes	Yes	Yes	Low	Include
Influence of blood group, Glucose-6-phosphate dehydrogenase and Haemoglobin genotype on Falciparum malaria in children in Vihiga highland of Western Kenya.	2020	Yes	Yes	Yes	Yes	Yes	Yes	Yes	Yes	Yes	Low	Include
Influence of haemoglobins S and C on predominantly asymptomatic Plasmodium infections in northern Ghana.	2010	Yes	Yes	Yes	Yes	Yes	Yes	Yes	Yes	Yes	Low	Include
Influence of hemoglobinopathies and glucose-6-	2020	Yes	Yes	Yes	Yes	Yes	Yes	Yes	Yes	Yes	Low	Include

phosphate dehydrogenase deficiency on diagnosis of diabetes by HbA1c among Tanzanian adults with and without HIV: A cross-sectional study.												
Inherited hemoglobin disorders in Guinea-Bissau, West Africa: a population study.	2006	Yes	Yes	Yes	Yes	Yes	Yes	Yes	Yes	Yes	Low	Include
Malaria as a cause of morbidity and mortality in children with homozygous sickle cell disease on the coast of Kenya.	2009	Yes	Yes	Yes	Yes	Yes	Yes	Yes	Yes	Yes	Low	Include
Modification in the frequency of Hb C and Hb S in Burkina Faso: an influence of migratory fluxes and improvement of patient health care.	2002	Yes	Yes	Yes	Yes	Yes	Yes	Yes	Yes	Yes	Low	Include
Neonatal haemoglobinopathy screening in Burkina Faso.	2009	Yes	Yes	Yes	Yes	Yes	Yes	Yes	Yes	Yes	Low	Include
Neonatal screening for sickle cell anaemia in the Democratic Republic of the Congo: experience from a pioneer project on 31 204 newborns.	2009	Yes	Yes	Yes	Yes	Yes	Yes	Yes	Yes	Yes	Low	Include
Newborn screening for sickle cell disease at Kisumu county hospital, Kisumu – Kenya	2019	Yes	Yes	Yes	Yes	Yes	Yes	Yes	Yes	Yes	Low	Include
Newborn screening for sickle cell disease	2008	Yes	Yes	Yes	Yes	Yes	Yes	Yes	Yes	Yes	Low	Include

in a Nigerian hospital.												
Newborn screening for sickle cell disease in Butembo and Beni: a pilot experience in a highland region of the Democratic Republic of Congo.	2023	Yes	Yes	Yes	Yes	Yes	Yes	Yes	Yes	Yes	Low	Include
Newborn Screening for Sickle Cell Disease in Liberia: A Pilot Study.	2016	Yes	Yes	Yes	Yes	Yes	Yes	Yes	Yes	Yes	Low	Include
Newborn screening for sickle cell disease in the Republic of Benin.	2009	Yes	Yes	Yes	Yes	Yes	Yes	Yes	Yes	Yes	Low	Include
Newborn screening for sickle cell disease: an innovative pilot program to improve child survival in Dar es Salaam, Tanzania.	2019	Yes	Yes	Yes	Yes	Yes	Yes	Yes	Yes	Yes	Low	Include
Pattern of haemoglobin phenotypes in newborn infants at the national hospital abuja using high performance liquid chromatography.	2020	Yes	Yes	Yes	Yes	Yes	Yes	Yes	Yes	Yes	Low	Include
Point-of-Care Newborn Screening for Sickle Cell Disease at Selected Health Facilities in the Gambia.	2024	Yes	Yes	Yes	Yes	Yes	Yes	Yes	Yes	Yes	Low	Include
Point-of-care testing allows successful simultaneous screening of sickle cell disease, HIV, and tuberculosis for households in rural	2022	Yes	Yes	Yes	Yes	Yes	Yes	Yes	Yes	Yes	Low	Include

Guinea-Bissau, West Africa.												
Potential of point of care tests for newborn screening for sickle cell disease: Evaluation of HemotypeSC™ and sickle SCAN® in Tanzania.	2022	Yes	Yes	Yes	Yes	Yes	Yes	Yes	Yes	Yes	Low	Include
Prevalence and impact of sickle cell trait on the clinical and laboratory parameters of HIV infected children in Lagos, Nigeria.	2018	Yes	Yes	Yes	Yes	Yes	Yes	Yes	Yes	Yes	Low	Include
Prevalence and pattern of sickle cell disease in premarital couples in Southeastern Nigeria.	2013	Yes	Yes	Yes	Yes	Yes	Yes	Yes	Yes	Yes	Low	Include
Prevalence of anaemia at booking in a semi-urban community in north-central Nigeria.	2014	Yes	Yes	Yes	Yes	Yes	Yes	Yes	Yes	Yes	Low	Include
Prevalence of classic erythrocyte polymorphisms among 749 children in southern highland Rwanda.	2012	Yes	Yes	Yes	Yes	Yes	Yes	Yes	Yes	Yes	Low	Include
Prevalence of hemoglobin abnormalities in an apparently healthy population in Benin.	2020	Yes	Yes	Yes	Yes	Yes	Yes	Yes	Yes	Not Applicable	Low	Include
Prevalence of Hemoglobin-S and Baseline Level of Knowledge on Sickle Cell Disease Among Pregnant Women	2022	Yes	Yes	Yes	Yes	Yes	Yes	Yes	Yes	Yes	Low	Include

Attending Antenatal Clinics in Dar-Es-Salaam, Tanzania.												
Prevalence of inherited blood disorders and associations with malaria and anemia in Malawian children.	2018	Yes	Yes	Yes	Yes	Yes	Yes	Yes	Yes	Yes	Low	Include
Prevalence of Inherited Hemoglobin Disorders and Relationships with Anemia and Micronutrient Status among Children in Yaoundé and Douala, Cameroon.	2017	Yes	Yes	Yes	Yes	Yes	Yes	Yes	Yes	Yes	Low	Include
Prevalence of Plasmodium falciparum and non-P. falciparum infections in a highland district in Ghana, and the influence of HIV and sickle cell disease.	2017	Yes	Yes	Yes	Yes	Yes	Yes	Yes	Yes	Yes	Low	Include
Prevalence of polymorphisms in glucose-6-phosphate dehydrogenase, sickle haemoglobin and nitric oxide synthase genes and their relationship with incidence of uncomplicated malaria in Iganga, Uganda.	2017	Yes	Yes	Yes	Yes	Yes	Yes	Yes	Yes	Yes	Low	Include
Prevalence of sickle cell among patients attending immunogenetic diagnostic laboratory	2018	Yes	Yes	Yes	Yes	Yes	Yes	Yes	Yes	Yes	Low	Include

Akwanga, Nasarawa state Nigeria												
Prevalence of sickle cell and α-Thalassemia traits in children enrolled in a Malaria vaccine clinical trial, in Kombewa, Western Kenya	2007	Yes	Yes	Yes	Yes	Yes	Yes	Yes	Yes	Yes	Low	Include
Prevalence of sickle cell disease and other haemoglobin variants in Calabar, Cross River State, Nigeria	2019	Yes	Yes	Yes	Yes	Yes	Yes	Yes	Yes	Not Applicable	Low	Include
Prevalence of sickle cell disease in a northeastern region of the Democratic Republic of Congo: what impact on transfusion policy?	2010	Yes	Yes	Yes	Yes	Yes	Yes	Yes	Yes	Yes	Low	Include
Prevalence of sickle cell disease in a pediatric population suffering from severe infections: a Congolese experience.	2014	Yes	Yes	Yes	Yes	Yes	Yes	Yes	Yes	Yes	Low	Include
Prevalence Of Sickle Cell Disease In Kilamba Kiaxi Hospital, Luanda, Angola	2012	Yes	Yes	Yes	Yes	Yes	Yes	Yes	Yes	Not Applicable	Low	Include
Prevalence of sickle cell disorders and malaria infection in children aged 1-12 years in the Volta Region, Ghana: a community-based study.	2020	Yes	Yes	Yes	Yes	Yes	Yes	Yes	Yes	Yes	Low	Include
Prevalence of sickle cell gene among	2016	Yes	Yes	Yes	Yes	Yes	Yes	Yes	Yes	Yes	Low	Include

apparently healthy under-two south-east Nigerian children: what is the role of parental premarital counselling and socio-demographic characteristics? A pilot study.												
Prevalence of sickle cell trait and its association to renal dysfunction among blood donors at University of Medical Sciences Teaching Hospital, Ondo, Nigeria.	2021	Yes	Yes	Yes	Yes	Yes	Yes	Yes	Yes	Yes	Low	Include
Prevalence of sickle cell trait and needs assessment for uptake of sickle cell screening among secondary school students in Kampala City, Uganda.	2024	Yes	Yes	Yes	Yes	Yes	Yes	Yes	Yes	Yes	Low	Include
Prevalence of Sickle Cell Trait and Reliability of Self-Reported Status among Expectant Parents in Nigeria: Implications for Targeted Newborn Screening.	2016	Yes	Yes	Yes	Yes	Yes	Yes	Yes	Yes	Yes	Low	Include
Prevalence of the sickle cell trait in Gabon: a nationwide study.	2014	Yes	Yes	Yes	Yes	Yes	Yes	Yes	Yes	Yes	Low	Include
Prospective Newborn Screening for Sickle Cell Disease and Other Inherited Blood Disorders in Central Malawi.	2021	Yes	Yes	Yes	Yes	Yes	Yes	Yes	Yes	Not Applicable	Low	Include

Risk factors for recurrent severe anemia among previously transfused children in Uganda: an age-matched case-control study.	2019	Yes	Yes	Yes	Yes	Yes	Yes	Yes	Yes	Not Applicable	Low	Include
Routine hemoglobin electrophoresis for pediatric surgery day case in at-risk populations: Suggested algorithm for screening using clinical risk factors.	2016	Yes	Yes	Yes	Yes	Yes	Yes	Yes	Yes	Yes	Low	Include
Sickle cell anaemia: a prevalence study among the children attending Usmanu Danfodiyo University Teaching Hospital, Sokoto, North-Western Nigeria	2017	Yes	Yes	Yes	Yes	Yes	Yes	Yes	Yes	Yes	Low	Include
Sickle cell disease: caregiver's awareness and phenotype distribution among children presenting to children emergency of a tertiary hospital in Lagos, Nigeria	2022	Yes	Yes	Yes	Yes	Yes	Yes	Yes	Yes	Yes	Low	Include
Sickle cell screening in Uganda: High burden, human immunodeficiency virus comorbidity, and genetic modifiers.	2019	Yes	Yes	Yes	Yes	Yes	Yes	Yes	Yes	Yes	Low	Include
Sickle cell trait, APOL1 risk allele status and chronic kidney disease among ART-experienced adults	2024	Yes	Yes	Yes	Yes	Yes	Yes	Yes	Yes	Yes	Low	Include

living with HIV in northern Nigeria.											
Sickle-cell disease in febrile children living in a rural village of Madagascar and association with malaria and respiratory infections.	2016	Yes	Yes	Yes	Yes	Yes	Yes	Yes	Yes	Yes	Low	Include
Surveillance for sickle cell disease, United Republic of Tanzania.	2020	Yes	Yes	Yes	Yes	Yes	Yes	Yes	Yes	Yes	Low	Include
The burden and consequences of inherited blood disorders among young children in western Kenya.	2014	Yes	Yes	Yes	Yes	Yes	Yes	Yes	Yes	Yes	Low	Include
The efficacy of maternal health education and maternal screening on knowledge and the uptake of infant screening for sickle cell disease in Dar-Es-Salaam, Tanzania; a quasi experimental study.	2023	Yes	Yes	Yes	Yes	Yes	Yes	Yes	Yes	Yes	Low	Include
The epidemiology of childhood sickle cell anaemia and the disease modifying effects of the -α3.7 deletional form of α-thalassaemia and the glucose-6-phosphate dehydrogenase G202T allele in Uganda ...	2024	Yes	Yes	Yes	Yes	Yes	Yes	Yes	Yes	Yes	Low	Include
The epidemiology of sickle cell disease in children recruited in	2019	Yes	Yes	Yes	Yes	Yes	Yes	Yes	Yes	Yes	Low	Include

infancy in Kilifi, Kenya: a prospective cohort study												
The feasibility of establishing sickle cell disease screening services at health centers in Uganda	2009	Yes	Yes	Yes	Yes	Yes	Yes	Yes	Yes	Yes	Low	Include
The Pattern of Haemoglobin Phenotypes and the Clinical Identity of Clients Tested at a Private Diagnostic Center in South-West Nigeria.	2021	Yes	Yes	Yes	Yes	Yes	Yes	Yes	Yes	Not Applicable	Low	Include
The Pattern of Sickle Cell Disease in Sickle Cell Patients from Northwestern Nigeria	2016	Yes	Yes	Yes	Yes	Yes	Yes	Yes	Yes	Not Applicable	Low	Include
The population dynamics of hemoglobins A, A(2), F and S in the context of the hemoglobinopathies HbS and α-thalassemia in Kenyan infants.	2019	Yes	Yes	Yes	Yes	Yes	Yes	Yes	Yes	Yes	Low	Include
The prevalence of sickle cell trait among adolescents in Bungoma County, Kenya (Doctoral dissertation, Moi University)	2020	Yes	Yes	Yes	Yes	Yes	Yes	Yes	Yes	Yes	Low	Include
The rate and pattern of fetal hemoglobin decline adjusted to sickle cell status of newborns in Dar es Salaam, Tanzania: A prospective cohort study.	2023	Yes	Yes	Yes	Yes	Yes	Yes	Yes	Yes	Yes	Low	Include

Unusual β-Globin Haplotype Distribution in Newborns from Bengo, Angola.	2019	Yes	Yes	Yes	Yes	Yes	Yes	Yes	Yes	Yes	Low	Include
[Epidemiology and prognostic factors for sickle cell retinopathy in Ouagadougou (Burkina Faso)].	2009	Yes	Un Clear	Yes	Yes	Yes	Yes	Yes	Yes	Not Applicable	Moderate	Include
[Rheumatologic diseases and haemoglobinopathies in Ouagadougou (Burkina Faso)].	2010	Yes	Un Clear	Yes	Yes	Yes	Yes	Yes	Yes	Not Applicable	Moderate	Include
[Strokes and hemoglobinopathies in Burkina Faso].	2012	No	Yes	No	Yes	Yes	Yes	Yes	Yes	Yes	Moderate	Include
Epidemiological profile of hemoglobinopathies in the Mauritanian population.	2002	No	Yes	Yes	Yes	Yes	Yes	Yes	Yes	Yes	Moderate	Include
Glucose-6-Phosphate Dehydrogenase Deficiency and Sickle Cell Trait among Prospective Blood Donors: A Cross-Sectional Study in Berekum, Ghana.	2016	No	Yes	Yes	Yes	Yes	Yes	Yes	Yes	Yes	Moderate	Include
Hemoglobinopathies, merozoite surface protein-2 gene polymorphisms, and acquisition of Epstein Barr virus among infants in Western Kenya.	2023	Yes	Un Clear	Un Clear	Yes	Yes	Yes	Yes	Yes	Yes	Moderate	Include
Prevalence of Sickle Cell Disease Among Anaemic Children Attending Mbeya	2018	Yes	Un Clear	No	Yes	Yes	Yes	Yes	Yes	Yes	Moderate	Include

Referral Hospital in Southern Tanzania.												
Routine haemoglobin electrophoresis screening in day case herniotomy in Nigerian children: Is it evidence-based?	2013	Yes	Yes	No	Yes	Yes	Yes	Yes	Yes	Yes	Moderate	Include
Routine Screening for Sickle Cell Disease during Pregnancy: Epidemiological and Haemoglobin Profile in Congo.	2022	Yes	Un Clear	Yes	Yes	Yes	Yes	Yes	Yes	Yes	Moderate	Include
Trends in sickle cell trait and disease screening in the Republic of Uganda, 2014-2019.	2021	Yes	Not Applicable	Yes	Yes	Yes	Yes	Yes	Yes	Yes	Moderate	Include
