# Supplementary material for: Burden of sickle cell anemia in Africa: A systematic review and meta-analysis
Source: PLoS One. 2025 Nov 25;20(11):e0337090. doi: 10.1371/journal.pone.0337090 (PMC12646443; doi:10.1371/journal.pone.0337090)
Supplement: S5 Table — (PDF) [file pone.0337090.s005.pdf]

S5 Table. Results of the Leave-Out-One sensitivity analysis. pdf

Study	Original_Prevalence	Original_CI	Prevalence_Without_Study	CI_Without_Study	Absolute_Difference	Percentage_Change	Influential
Omitting Arielle et al (2021)	0.0143	0.0108 - 0.0188	0.0136	0.0102 - 0.018	7.00E-04	1.18	No
Omitting Ezenwosu et al (2021)	0.0143	0.0108 - 0.0188	0.0147	0.0112 - 0.0193	5.00E-04	0.76	No
Omitting Saganuwan et al (2016)	0.0143	0.0108 - 0.0188	0.0138	0.0106 - 0.0181	4.00E-04	0.7	No
Omitting Nisreen et al (2010)	0.0143	0.0108 - 0.0188	0.0139	0.0105 - 0.0182	4.00E-04	0.68	No
Omitting Tegha et al (2021)	0.0143	0.0108 - 0.0188	0.0146	0.0111 - 0.0193	4.00E-04	0.62	No
Omitting Simpore et al (2002)	0.0143	0.0108 - 0.0188	0.0146	0.0111 - 0.0192	4.00E-04	0.61	No
Omitting Musyoka et al (2018)	0.0143	0.0108 - 0.0188	0.0139	0.0105 - 0.0183	4.00E-04	0.62	No
Omitting Abdala et al (2021)	0.0143	0.0108 - 0.0188	0.0139	0.0106 - 0.0183	3.00E-04	0.55	No
Omitting Abdala et al (2024)	0.0143	0.0108 - 0.0188	0.0139	0.0106 - 0.0183	3.00E-04	0.55	No
Omitting burnham et al (2016)	0.0143	0.0108 - 0.0188	0.0146	0.0111 - 0.0192	3.00E-04	0.53	No
Omitting Jiya et al (2017)	0.0143	0.0108 - 0.0188	0.0139	0.0106 - 0.0184	3.00E-04	0.53	No
Omitting Kosiyo et al (2020)	0.0143	0.0108 - 0.0188	0.0139	0.0106 - 0.0184	3.00E-04	0.53	No
Omitting Dodo et al (2018)	0.0143	0.0108 - 0.0188	0.0139	0.0106 - 0.0183	3.00E-04	0.52	No
Omitting Simpore et al (2007)	0.0143	0.0108 - 0.0188	0.0146	0.011 - 0.0192	3.00E-04	0.5	No
Omitting Abdulhamid et al (2024)	0.0143	0.0108 - 0.0188	0.0146	0.0111 - 0.0191	3.00E-04	0.5	No
Omitting Oppong et al (2020)	0.0143	0.0108 - 0.0188	0.014	0.0106 - 0.0184	3.00E-04	0.51	No
Omitting Anabire et al (2018)	0.0143	0.0108 - 0.0188	0.014	0.0106 - 0.0184	3.00E-04	0.49	No
Omitting Nankanja et al (2019)	0.0143	0.0108 - 0.0188	0.014	0.0106 - 0.0184	3.00E-04	0.47	No
Omitting Brahim et al (2024)	0.0143	0.0108 - 0.0188	0.014	0.0106 - 0.0184	3.00E-04	0.47	No
Omitting Kondani et al (2014)	0.0143	0.0108 - 0.0188	0.014	0.0106 - 0.0185	3.00E-04	0.43	No
Omitting Dhabangi et al (2019)	0.0143	0.0108 - 0.0188	0.014	0.0106 - 0.0185	3.00E-04	0.43	No
Omitting Lopera-Mesa et al (2015)	0.0143	0.0108 - 0.0188	0.0145	0.011 - 0.0191	3.00E-04	0.42	No
Omitting Alagbe et al (2021)	0.0143	0.0108 - 0.0188	0.014	0.0106 - 0.0185	2.00E-04	0.42	No
Omitting Loembet et al (2014)	0.0143	0.0108 - 0.0188	0.0145	0.011 - 0.019	2.00E-04	0.4	No

Omitting Mumbere et al (2023)	0.0143	0.0108 - 0.0188	0.0145	0.011 - 0.0191	2.00E-04	0.37	No
Omitting Oluwole et al (2022)	0.0143	0.0108 - 0.0188	0.014	0.0106 - 0.0185	2.00E-04	0.37	No
Omitting Masmas et al (2006)	0.0143	0.0108 - 0.0188	0.0145	0.011 - 0.0191	2.00E-04	0.35	No
Omitting MacGann et al (2018)	0.0143	0.0108 - 0.0188	0.0145	0.011 - 0.019	2.00E-04	0.35	No
Omitting Danquah et al (2010)	0.0143	0.0108 - 0.0188	0.0145	0.0109 - 0.0191	2.00E-04	0.34	No
Omitting Nyangasa et al (2023)	0.0143	0.0108 - 0.0188	0.0141	0.0106 - 0.0185	2.00E-04	0.35	No
Omitting Zohoun et al (2020)	0.0143	0.0108 - 0.0188	0.0144	0.011 - 0.019	2.00E-04	0.31	No
Omitting Gahutu et al (2012)	0.0143	0.0108 - 0.0188	0.0144	0.0109 - 0.019	2.00E-04	0.3	No
Omitting Kiyaga Charles (2023)	0.0143	0.0108 - 0.0188	0.0144	0.0109 - 0.019	2.00E-04	0.28	No
Omitting Menzato et al (2022)	0.0143	0.0108 - 0.0188	0.0144	0.0109 - 0.019	2.00E-04	0.27	No
Omitting Wirth et al (2019)	0.0143	0.0108 - 0.0188	0.0141	0.0107 - 0.0186	2.00E-04	0.27	No
Omitting Asare et al (2024)	0.0143	0.0108 - 0.0188	0.0144	0.0109 - 0.019	2.00E-04	0.25	No
Omitting Deyde et al (2002)	0.0143	0.0108 - 0.0188	0.0144	0.0109 - 0.019	2.00E-04	0.25	No
Omitting Moormann et al (2003)	0.0143	0.0108 - 0.0188	0.0144	0.0109 - 0.019	2.00E-04	0.25	No
Omitting Jeremiah et al (2006)	0.0143	0.0108 - 0.0188	0.0144	0.0109 - 0.019	1.00E-04	0.24	No
Omitting Tchum et al (2023)	0.0143	0.0108 - 0.0188	0.0144	0.0109 - 0.019	1.00E-04	0.24	No
Omitting Gomez et al (2024)	0.0143	0.0108 - 0.0188	0.0141	0.0107 - 0.0186	1.00E-04	0.24	No
Omitting Christopher et al (2022)	0.0143	0.0108 - 0.0188	0.0144	0.0109 - 0.019	1.00E-04	0.23	No
Omitting Komab et al (2009)	0.0143	0.0108 - 0.0188	0.0141	0.0107 - 0.0187	1.00E-04	0.23	No
Omitting Lwanira et al (2017)	0.0143	0.0108 - 0.0188	0.0144	0.0109 - 0.019	1.00E-04	0.23	No
Omitting Kuta et al (2019)	0.0143	0.0108 - 0.0188	0.0141	0.0107 - 0.0187	1.00E-04	0.23	No
Omitting Borges et al (2019)	0.0143	0.0108 - 0.0188	0.0141	0.0107 - 0.0187	1.00E-04	0.22	No
Omitting Adeboye et al (2016)	0.0143	0.0108 - 0.0188	0.0141	0.0107 - 0.0187	1.00E-04	0.21	No
Omitting Kweka et al (2020)	0.0143	0.0108 - 0.0188	0.0144	0.0109 - 0.019	1.00E-04	0.21	No
Omitting Fenomanana et al (2020)	0.0143	0.0108 - 0.0188	0.0144	0.0109 - 0.019	1.00E-04	0.21	No
Omitting McGann et al (2013)	0.0143	0.0108 - 0.0188	0.0141	0.0107 - 0.0187	1.00E-04	0.21	No
Omitting Eastburg (2020)	0.0143	0.0108 - 0.0188	0.0141	0.0107 - 0.0187	1.00E-04	0.2	No
Omitting Hernandez et al (2021)	0.0143	0.0108 - 0.0188	0.0141	0.0107 - 0.0187	1.00E-04	0.2	No
Omitting Tutuba et al (2022)	0.0143	0.0108 - 0.0188	0.0144	0.0109 - 0.019	1.00E-04	0.19	No

Omitting Hernandez et al (2016)	0.0143	0.0108 - 0.0188	0.0144	0.0109 - 0.019	1.00E-04	0.18	No
Omitting Nacoulma et al (2007)	0.0143	0.0108 - 0.0188	0.0141	0.0107 - 0.0187	1.00E-04	0.18	No
Omitting Tossea et al (2018)	0.0143	0.0108 - 0.0188	0.0144	0.0109 - 0.019	1.00E-04	0.18	No
Omitting Odunvbun et al (2008)	0.0143	0.0108 - 0.0188	0.0141	0.0107 - 0.0187	1.00E-04	0.18	No
Omitting Aluoch (1997)	0.0143	0.0108 - 0.0188	0.0142	0.0107 - 0.0187	1.00E-04	0.18	No
Omitting Uedraogo et al (2010)	0.0143	0.0108 - 0.0188	0.0144	0.0109 - 0.0189	1.00E-04	0.17	No
Omitting Oluwole et al (2020)	0.0143	0.0108 - 0.0188	0.0144	0.0109 - 0.0189	1.00E-04	0.16	No
Omitting Ndila (2019)	0.0143	0.0108 - 0.0188	0.0144	0.0109 - 0.019	1.00E-04	0.16	No
Omitting Watenga et al (2019)	0.0143	0.0108 - 0.0188	0.0143	0.0109 - 0.0189	1.00E-04	0.16	No
Omitting Kambale Kombi et al (2022)	0.0143	0.0108 - 0.0188	0.0143	0.0109 - 0.0189	1.00E-04	0.15	No
Omitting Nkya et al (2019)	0.0143	0.0108 - 0.0188	0.0143	0.0108 - 0.019	1.00E-04	0.15	No
Omitting Simpore et al (2002)	0.0143	0.0108 - 0.0188	0.0142	0.0107 - 0.0187	1.00E-04	0.15	No
Omitting Ada et al (2018)	0.0143	0.0108 - 0.0188	0.0142	0.0107 - 0.0187	1.00E-04	0.15	No
Omitting Ahmed et al (2020)	0.0143	0.0108 - 0.0188	0.0143	0.0109 - 0.0189	1.00E-04	0.15	No
Omitting David et al (2018)	0.0143	0.0108 - 0.0188	0.0143	0.0109 - 0.0189	1.00E-04	0.15	No
Omitting Ndeezi 2016 (2016)	0.0143	0.0108 - 0.0188	0.0143	0.0108 - 0.0189	1.00E-04	0.15	No
Omitting Mashingaidze et al (2024)	0.0143	0.0108 - 0.0188	0.0143	0.0109 - 0.0189	1.00E-04	0.15	No
Omitting Adu et al (2016)	0.0143	0.0108 - 0.0188	0.0143	0.0109 - 0.0189	1.00E-04	0.15	No
Omitting Macharia et al (2019)	0.0143	0.0108 - 0.0188	0.0143	0.0108 - 0.0189	1.00E-04	0.14	No
Omitting Millimono et al (2011)	0.0143	0.0108 - 0.0188	0.0143	0.0109 - 0.0189	1.00E-04	0.14	No
Omitting Kingsley et al (2019)	0.0143	0.0108 - 0.0188	0.0142	0.0107 - 0.0187	1.00E-04	0.14	No
Omitting Wegmuller et al (2020)	0.0143	0.0108 - 0.0188	0.0143	0.0108 - 0.0189	1.00E-04	0.13	No
Omitting Maeder et al (2016)	0.0143	0.0108 - 0.0188	0.0142	0.0107 - 0.0187	1.00E-04	0.14	No
Omitting Mockenhaupt et al (2000)	0.0143	0.0108 - 0.0188	0.0143	0.0108 - 0.0189	1.00E-04	0.13	No
Omitting Uyoga et al (2019)	0.0143	0.0108 - 0.0188	0.0143	0.0108 - 0.0189	1.00E-04	0.13	No
Omitting Tshilolo (2008)	0.0143	0.0108 - 0.0188	0.0142	0.0107 - 0.0187	1.00E-04	0.13	No
Omitting Namukasa et al (2024)	0.0143	0.0108 - 0.0188	0.0143	0.0108 - 0.0189	1.00E-04	0.12	No
Omitting Mpimbaza et al (2018)	0.0143	0.0108 - 0.0188	0.0143	0.0108 - 0.0189	1.00E-04	0.12	No

Omitting Rahimy et al (2008)	0.0143	0.0108 - 0.0188	0.0142	0.0107 - 0.0187	1.00E-04	0.12	No
Omitting Musoriza et al (2007)	0.0143	0.0108 - 0.0188	0.0143	0.0108 - 0.0189	1.00E-04	0.11	No
Omitting Kafando et al (2008)	0.0143	0.0108 - 0.0188	0.0142	0.0107 - 0.0188	1.00E-04	0.11	No
Omitting Amoako et al (2014)	0.0143	0.0108 - 0.0188	0.0143	0.0108 - 0.0189	1.00E-04	0.09	No
Omitting Akinbodewa et al (2021)	0.0143	0.0108 - 0.0188	0.0143	0.0108 - 0.0189	1.00E-04	0.09	No
Omitting Ngwengi et al (2020)	0.0143	0.0108 - 0.0188	0.0143	0.0108 - 0.0189	1.00E-04	0.09	No
Omitting Nnaji et al (2013)	0.0143	0.0108 - 0.0188	0.0143	0.0108 - 0.0189	1.00E-04	0.09	No
Omitting Agasa et al (2009)	0.0143	0.0108 - 0.0188	0.0143	0.0108 - 0.0189	1.00E-04	0.09	No
Omitting Owusu et al (2017)	0.0143	0.0108 - 0.0188	0.0143	0.0108 - 0.0189	1.00E-04	0.09	No
Omitting Ademuyiwa et al (2016)	0.0143	0.0108 - 0.0188	0.0143	0.0108 - 0.0189	0	0.07	No
Omitting Okocha et al (2016)	0.0143	0.0108 - 0.0188	0.0143	0.0108 - 0.0189	0	0.07	No
Omitting Nafiu et al (2020)	0.0143	0.0108 - 0.0188	0.0142	0.0107 - 0.0188	0	0.07	No
Omitting Oleweet al (2023)	0.0143	0.0108 - 0.0188	0.0143	0.0108 - 0.0189	0	0.07	No
Omitting Dokekias et al (2022)	0.0143	0.0108 - 0.0188	0.0142	0.0107 - 0.0188	0	0.06	No
Omitting Kiyaga et al (2019)	0.0143	0.0108 - 0.0188	0.0142	0.0107 - 0.0188	0	0.06	No
Omitting Halima et al (2023)	0.0143	0.0108 - 0.0188	0.0142	0.0107 - 0.0188	0	0.06	No
Omitting Umoh et al (2010)	0.0143	0.0108 - 0.0188	0.0142	0.0107 - 0.0188	0	0.05	No
Omitting Moez et al (2015)	0.0143	0.0108 - 0.0188	0.0142	0.0108 - 0.0188	0	0.05	No
Omitting Okwi et al (2010)	0.0143	0.0108 - 0.0188	0.0142	0.0108 - 0.0188	0	0.04	No
Omitting Englestone et al (2017)	0.0143	0.0108 - 0.0188	0.0142	0.0108 - 0.0188	0	0.04	No
Omitting Adewara et al (2014)	0.0143	0.0108 - 0.0188	0.0143	0.0108 - 0.0189	0	0.04	No
Omitting Suchdev et al (2014)	0.0143	0.0108 - 0.0188	0.0142	0.0108 - 0.0188	0	0.04	No
Omitting Tubman et al (2016)	0.0143	0.0108 - 0.0188	0.0143	0.0108 - 0.0189	0	0.04	No
Omitting Ademuyiwa et al (2013)	0.0143	0.0108 - 0.0188	0.0143	0.0108 - 0.0188	0	0.04	No
Omitting Lussiana et al (2021)	0.0143	0.0108 - 0.0188	0.0143	0.0109 - 0.0186	0	0.03	No
Omitting Islam et al (2021)	0.0143	0.0108 - 0.0188	0.0142	0.0108 - 0.0188	0	0.03	No
Omitting Tutuba et al (2023)	0.0143	0.0108 - 0.0188	0.0142	0.0108 - 0.0188	0	0.03	No
Omitting Ambrose et al (2020)	0.0143	0.0108 - 0.0188	0.0142	0.0108 - 0.0188	0	0.02	No
Omitting Okwi et al (2009)	0.0143	0.0108 - 0.0188	0.0143	0.0108 - 0.0189	0	0.02	No

Omitting Adegoke (2024)	0.0143	0.0108 - 0.0188	0.0143	0.0108 - 0.0189	0	0.02	No
Omitting Nwabuko et al (2020)	0.0143	0.0108 - 0.0188	0.0142	0.0108 - 0.0188	0	0.02	No
Omitting Nnodu et al (2020)	0.0143	0.0108 - 0.0188	0.0142	0.0108 - 0.0188	0	0.02	No
Omitting E.Ambrose et al (2017)	0.0143	0.0108 - 0.0188	0.0143	0.0108 - 0.0188	0	0	No
Omitting Khelili et al (2003)	0.0143	0.0108 - 0.0188	0.0143	0.0108 - 0.0188	0	0	No
Pooled estimate	0.0143	0.0108 - 0.0188	0.0143	0.0108 - 0.0188	0	0	No
	0.0143	0.0108 - 0.0188	NA	NA - NA	NA	NA	NA
