# Supplementary material for: Burden of sickle cell anemia in Africa: A systematic review and meta-analysis
Source: PLoS One. 2025 Nov 25;20(11):e0337090. doi: 10.1371/journal.pone.0337090 (PMC12646443; doi:10.1371/journal.pone.0337090)
Supplement: S1 Fig — (PDF) [file pone.0337090.s006.pdf]

S4 Fig. Certainty of evidence assessment using A Measurement Tool to Assess systematic Reviews (AMSTAR)

Reviews	1. PICO components[A]	2.* Pre-established protocol[B]	3. Explanation of included studies' design[A]	4.* Comprehensive search strategy[B]	5. Duplicate study selection[A]	6. Duplicate data extraction[A]	7.* List of excluded studies and justification[B]	8. Description of included studies[B]	9.* Assessment of RoB in included studies[B,C]	10. Funding sources[A]	11.* Use of appropriate statistical methods[C,D]	12. RoB impact on synthesized results[D]	13.* Results interpretation with RoB reference[A]	14. Heterogeneity explanation[A]	15.* Publication/ small study bias investigation[D]	16. Conflict of interest declaration[A]	Overall confidence[E]
	AMSTAR 2 items																
Prevalence of sickle cell Anemia in Africa: A systematic review and meta-analysis of studies 1994 – 2024	Yes	Yes	Yes	Yes	Yes	Yes	Yes	Yes	Partial Yes	No	Yes	Yes	Yes	Yes	Yes	Yes	High

AMSTAR 2, A MeaSurement Tool to Assess Systematic Reviews 2 (Shea et al. 2017; doi: 10.1136/bmj.j4008); PICO, participant, intervention, comparison, outcome; RoB, Risk of bias.

*Asterisk indicates a critical item (domain).

[A] Possible responses: Yes/No.

[B] Possible responses: Yes/Partial Yes/No. A 'Partial Yes' response is evaluated positively to the overall confidence rating.

[C] Item response depends on separate assessment of randomized controlled trials (RCTs) and non-randomized studies of healthcare interventions (NRSI) in the review.

[D] Possible responses: Yes/No/No MA. A 'No MA' response stands for 'no meta-analysis conducted' and does not affect the overall confidence rating.

[E] Critically Low/Low/Moderate/High confidence in the results of the review. Critically Low: more than one critical flaw with or without non-critical weaknesses, Low: One critical flaw with or without non-critical weaknesses, Moderate: No critical flaws but more than one non-critical weakness, High: No flaws at all or one non-critical weakness.
